# The *Drosophila*‐parasitizing wasp *Leptopilina heterotoma*: A comprehensive model system in ecology and evolution

**DOI:** 10.1002/ece3.9625

**Published:** 2023-01-23

**Authors:** Maude Quicray, Léonore Wilhelm, Thomas Enriquez, Shulin He, Mathilde Scheifler, Bertanne Visser

**Affiliations:** ^1^ Evolution and Ecophysiology Group, Department of Functional and Evolutionary Entomology University of Liège ‐ Gembloux Agro‐Bio Tech Gembloux Belgium

**Keywords:** associative learning, endosymbiont, fitness, host‐parasitoid community, lipids, sex pheromones, virulence

## Abstract

The parasitoid *Leptopilina heterotoma* has been used as a model system for more than 70 years, contributing greatly to diverse research areas in ecology and evolution. Here, we synthesized the large body of work on *L. heterotoma* with the aim to identify new research avenues that could be of interest also for researchers studying other parasitoids and insects. We start our review with a description of typical *L. heterotoma* characteristics, as well as that of the higher taxonomic groups to which this species belongs. We then continue discussing host suitability and immunity, foraging behaviors, as well as fat accumulation and life histories. We subsequently shift our focus towards parasitoid‐parasitoid interactions, including *L. heterotoma* coexistence within the larger guild of *Drosophila* parasitoids, chemical communication, as well as mating and population structuring. We conclude our review by highlighting the assets of *L. heterotoma* as a model system, including its intermediate life history syndromes, the ease of observing and collecting natural hosts and wasps, as well as recent genomic advances.

## Graphical abstract

The parasitoid *Leptopilina heterotoma* has been used as a model system in biology for more than 70 years. This review aims to provide a broad and detailed synthesis of the work performed on this system, including immunity, behavioral ecology, endosymbiotic and trophic interactions, as well as physiology. Overall, the scientific literature on *L. heterotoma* unites research based on field observations and experiments, as well as laboratory studies, highlighting the versatility of this model system.

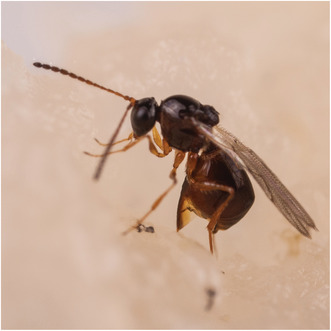

## 1 INTRODUCTION

**FIGURE 1:**
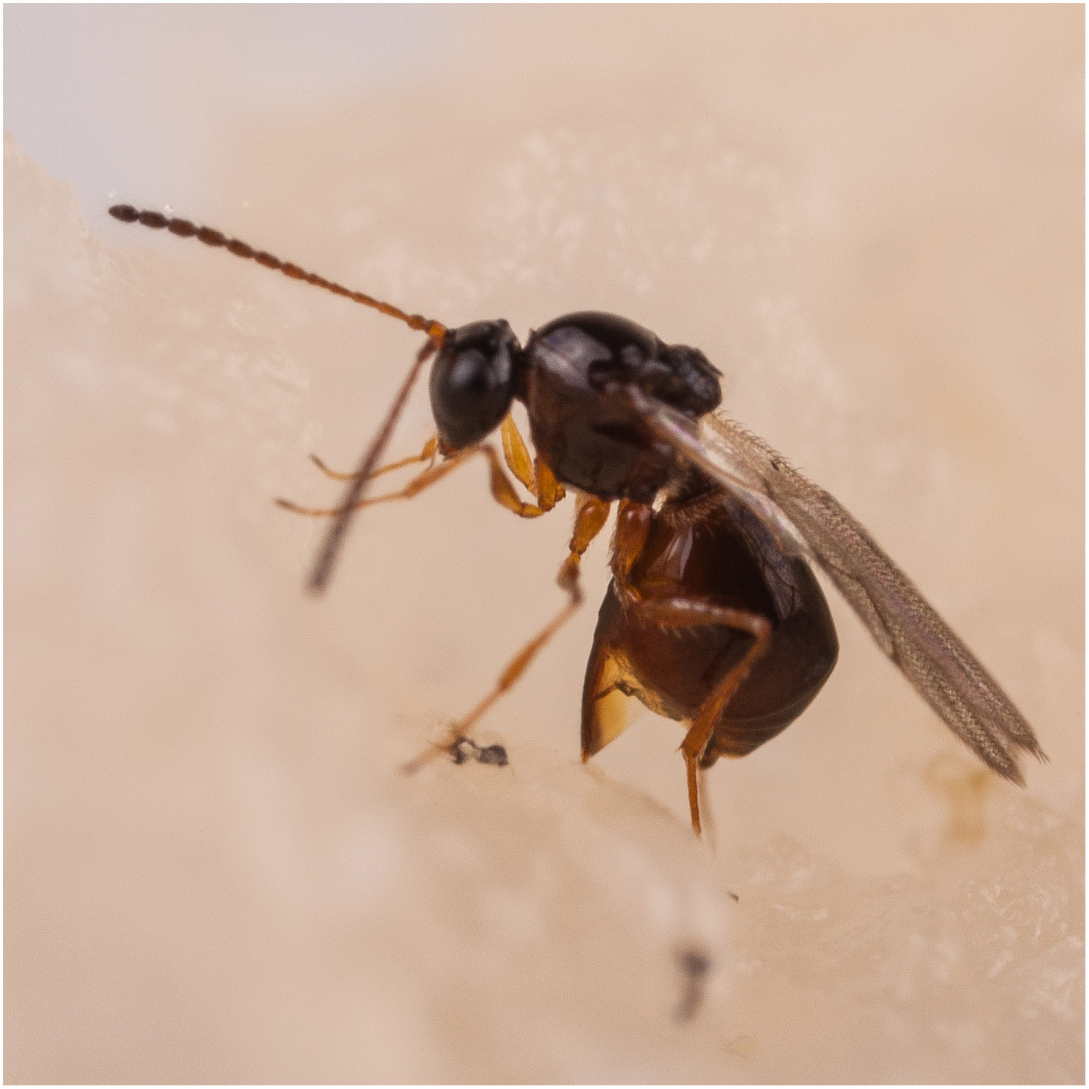
The amber wasp *Leptopilina heterotoma* © Hans Smidt

The parasitoid *Leptopilina heterotoma* is a wasp species that has long captivated biologists, with the earliest reports in the scientific literature dating back to the 1950s (Figure 1; Jenni, 1951). During the early days of scientific reporting, the species was often referred to as *Pseudeucoila bochei* (Weld, 1944), although *L. heterotoma (Thomson, 1862)* was the first recorded name for the species. This was highlighted by Nordlander (1980) in his comprehensive paper on the *Leptopilina* genus, which was recently updated by Lue et al. (2016) (including all other known synonyms of *L. heterotoma*; Table 1)*. L. heterotoma* belongs to the cynipoid wasps (superfamily: Cynipoidea), a group that contains parasitoids (i.e., insects that develop and feed on another insect; Godfray, 1994), but also includes phytophagous gall inducers and inquilines (i.e., inhabiting the galls of others). The wide diversity of feeding habits and life histories within the cynipoids has led to several hypotheses regarding the early evolution of the group. Ronquist (1995, 1999) hypothesized that the first cynipoids were endoparasitoids of wood‐, stem‐ or cone‐boring insect larvae. In a recent study by Blaimer et al. (2020) another scenario was proposed where inquilinism dominated throughout the early evolution of cynipoids. This means that cynipoids would be derived from gall‐associated inquiline ancestors. Another phylogenetic reconstruction, however, supported the previously suggested parasitoid‐first hypothesis, where the common ancestor of the cynipoids was a parasitoid. Irrespective of the exact lifestyle of the common ancestor, several host shifts have occurred in the cynipoids, including the use of dipteran hosts, as is the case for *L. heterotoma*.

**TABLE 1:** Synonyms of *L. heterotoma* (from Lue et al., 2016)

*Eucoila heterotoma*
*Ganaspis subnuda*
*Ganaspis monilicornis*
*Erisphagia philippinensis*
*Pseudeucoila* (*Pseudeucoila*) *bochei*
*Cothonaspis* (*Erisphagia*) *philippinensis*
*Pseudeucoila bochei*
*Leptopilina monilicornis*
*Leptopilina philippinensis*
*Leptopilina subnuda*
*Leptopilina bochei*

**FIGURE 2:**
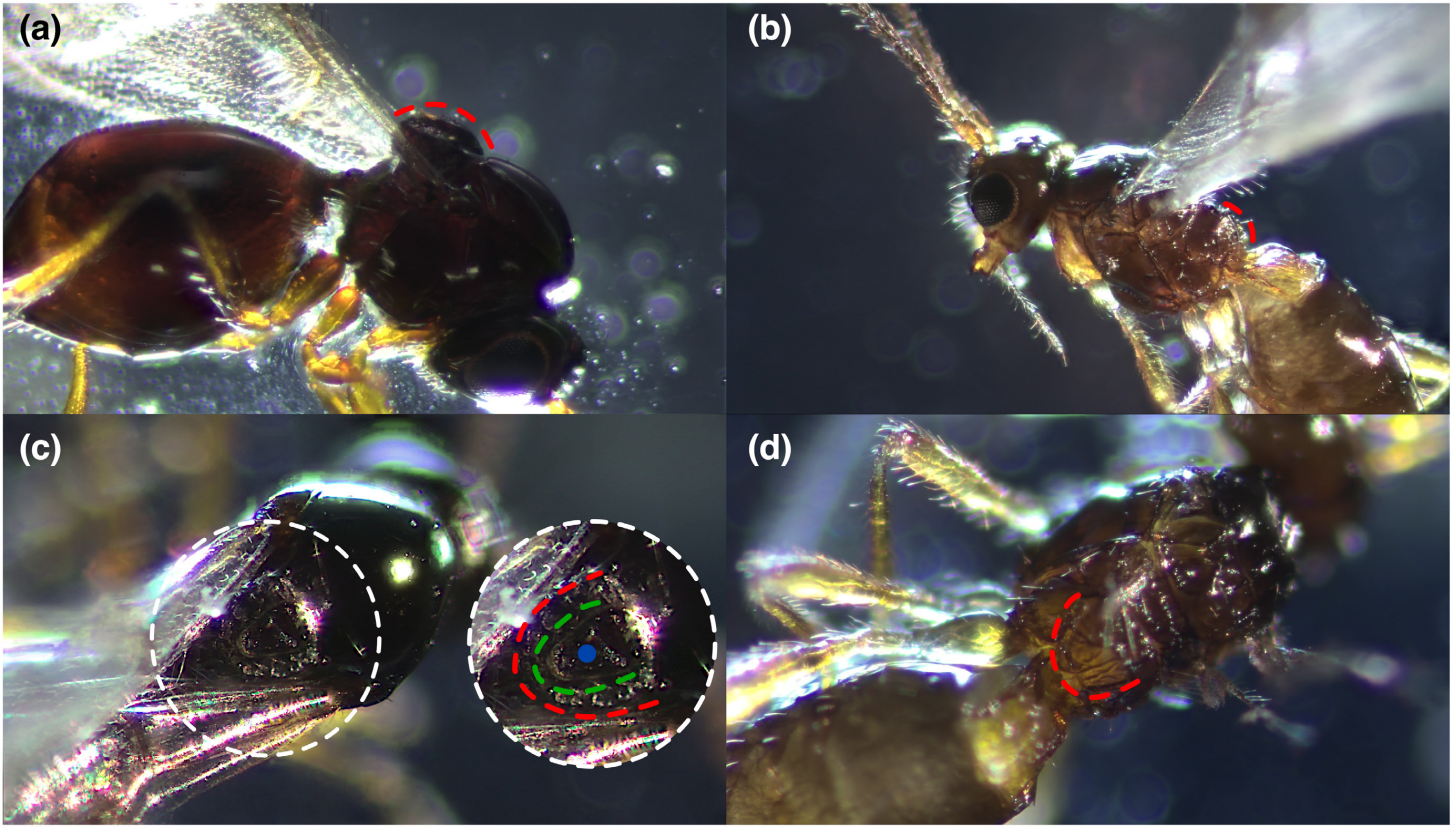
Lateral view of the thorax of *Leptopilina heterotoma* (a) and *Asobara tabida* (b) with the scutellum highlighted with a red line. Dorsal view of the scutellum for *L. heterotoma* (c) and *A. tabida* (d). The scutellar plate common to eucolines is highlighted with the green line, and the glandular pit with the blue dot.

*Leptopilina heterotoma* belongs to the figitid family (Figitidae) and the eucoline subfamily (Eucolinae). While delimitations of the figitids have not been well established, the eucoilines are easily identifiable by the possession of a clear synapomorphy: a scutellar plate with a glandular pit (with unknown function) surmounting the mesothoracic scutellum (Figure 2; Fontal‐Cazalla et al., 2002). Female antennae typically have 13 segments, while the male's antennae have 15 segments. Females also possess a clip at the end of their ovipositor, which is a unique feature of most figitid wasps in the subfamilies Figitinae and Eucolinae (see Section 5; Buffington, 2007). Eucoline adult sizes range from 1 to 5 mm, and the body, brown or black, is shiny. When a *L. heterotoma* individual is viewed from the side and in the light, the body appears to be amber colored. In trying to boost other researchers to work on *L. heterotoma*, and to ease the transfer of our scientific knowledge to the general public, we here propose “amber wasp” as the common name for the species.

**FIGURE 3:**
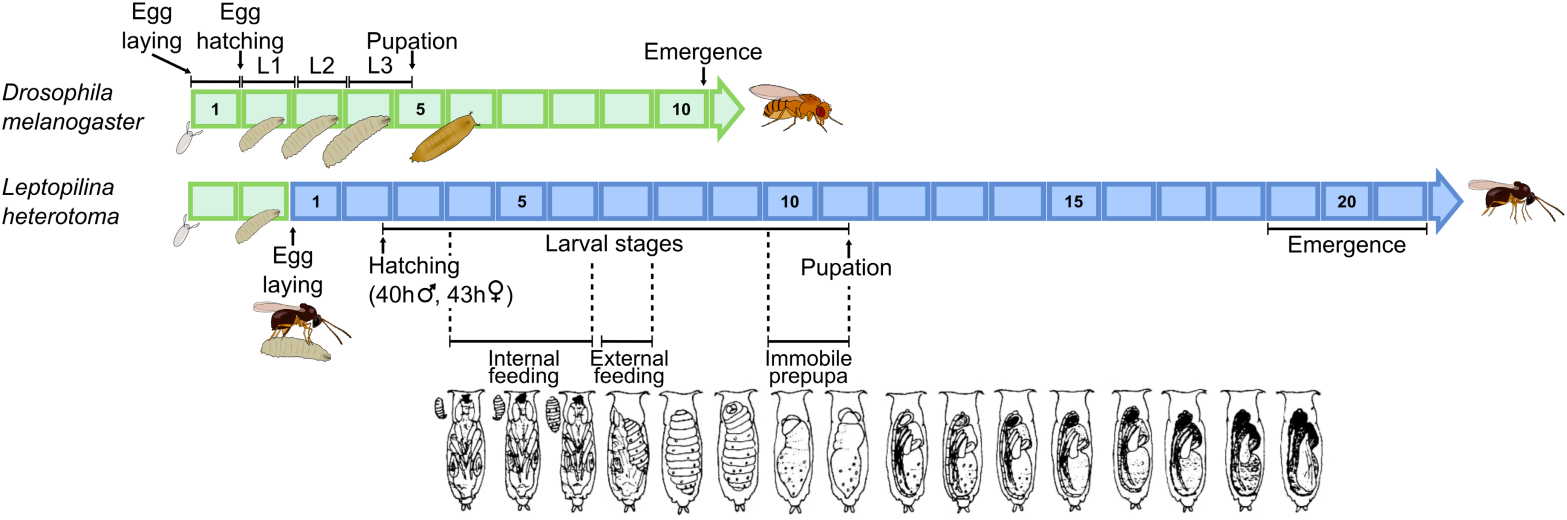
Development of *Leptopilina heterotoma*. Timeline of the developmental stages of *Drosophila melanogaster* (green), and *L. heterotoma* developing in *D. melanogaster* (blue) at 25°C. Numbers indicate the time in days (adapted from van Alphen & Thunnissen, 1983). *L. heterotoma* goes through three larval stages (Carton et al., 1986; Jenni, 1951) that may differ depending on the temperature and the host species used (Howe, 1967; Jenni, 1951). A female can oviposit in all larval host instars, but survival is highest when eggs are laid in second instar (Jenni, 1951). After ~30–34 h, the embryo possesses 10 segments (corresponding to the three thoracic and seven abdominal segments of the adult) that are clearly visible (Jenni, 1951). The egg then hatches after ~39–49 h, with females hatching approximately 3 h later than males (Eijsackers & Bakker, 1971). The first larval instar possesses caudal and thoracic appendages, and the larva uses its mandibles mainly to consume host hemolymph (Carton et al., 1986; Jenni, 1951). The first molt of the parasitoid takes place at approximately the same time as host pupation (Carton et al., 1986), which may have a similar hormonal basis (Kopelman & Chabora, 1984). From the second instar onwards, larvae use their mandibles to feed on the host's tissues (Carton et al., 1986). At the time of the second molt, the parasitoid leaves the host's body and lies in between the pupa and the puparium feeding as an ectoparasitoid (Carton et al., 1986). The third larval instar has a much rounder shape than the earlier instars and does not bear any appendages anymore. In the pre‐pupal stage, the larva loses its mandibles (Jenni, 1951) and excretes pellets (meconia) that become visible at the posterior end of the host puparium (Carton et al., 1986; Jenni, 1951). Pupation lasts approximately 9 days (Jenni, 1951) and the parasitoid becomes gradually pigmented (Jenni, 1951; van Alphen & Thunnissen, 1983). The time of emergence is ~21 days after oviposition for males, and ~23 days for females. After emerging from its own puparium, the adult *L. heterotoma* remains within the host's puparium for ~24 h before emergence (van Alphen & Thunnissen, 1983).

All eucolines are endoparasitoids of cyclorraphous fly larvae and have a worldwide distribution, with exception of the poles (Buffington et al., 2020). *Leptopilina heterotoma* can parasitize a range of different host species, mainly in the *Drosophila* fly genus (see Section 2), which it attacks when the host itself is developing as a larva. An egg is laid inside the host and only a single individual can successfully survive into adulthood, even if multiple eggs are laid within the same host (i.e., *L. heterotoma* is a solitary parasitoid). *L. heterotoma* is a koinobiont, meaning that the host continues feeding and growing while the wasp is developing. Interestingly, *L. heterotoma* initially develops inside the host, but will migrate out of the host's body during later developmental stages (10 days after oviposition; Figure 3). Eucoilines are generally pro‐ovigenic (Ellers & Jervis, 2004), and while *L. heterotoma* is often referred to as being pro‐ovigenic (Carton et al., 1986; Haccou et al., 1991; Kimura, 2019), for most strains tested so far considerable egg numbers (sometimes more than 300) are matured during adult life even if some eggs are mature at emergence (Vayssade et al., 2012).

*Drosophila* parasitoids, including *L. heterotoma*, have been reviewed in the past, most extensively in the book chapters of Carton et al. (1986) and Fleury et al. (2009). More recently, Wertheim (2022) has synthesized the work on host‐parasitoid co‐evolution in the context of virulence and immunity, including *L. heterotoma*. No review has yet been dedicated solely to the wasp *L. heterotoma* that, together with several other species in the *Leptopilina* genus, has been a staple of research in ecology and evolution since the 1950s. With this review, we synthesize key findings obtained with *L. heterotoma* as a model system, highlighting the major contribution this species has made to research in ecology and evolution. We further suggest avenues for future research to enthuse others to use this intriguing species as a model system.

## 2 HOST SUITABILITY, HOST RESISTANCE, AND PARASITOID VIRULENCE

The amber wasp *L. heterotoma* predominantly parasitizes hosts in the *Drosophila* genus, a very diverse and rich taxon, but also other drosophilid species, such as *Zaprionus* flies (Table S1). *L. heterotoma* does, however, not perform equally well on all these species, due to differences in suitability, and species‐specific immune reactions. Following oviposition of a wasp, a parasitized host can indeed initiate an immune response in <48 h in an attempt to kill the wasp's egg (Mortimer, 2013; Nappi, 1975; Poyet et al., 2013). While ovipositing, the female will also inject venom fluids that can suppress the host's immune response to increase the chances of successful parasitoid development. Adaptations and counter‐adaptations in wasp virulence and host immunity leads to an evolutionary arms race that has been particularly well studied in parasitoids (Wertheim, 2022). The interactions between *L. heterotoma*, as well as *L. boulardi*, and their hosts has greatly contributed to our understanding of both insect immunity and venom evolution in parasitoids. Several reviews have already discussed this in great detail (Mortimer, 2013; Nappi, 2010; Poirié et al., 2009, 2014; Wertheim, 2022; Yang et al., 2020); hence here we emphasize the work done on host suitability, host immunity and *L. heterotoma* virulence.

### 2.1 Host species suitability and phenology

*Drosophila* species can feed on a wide variety of substrates, including fruits, flowers, tree sap, cacti and mushrooms, generally in a state of decay (Markow & O'Grady, 2008). *Drosophila* mostly feed on the microbial community associated with decaying substrates, in addition to the substrate itself (Markow & O'Grady, 2008). Generalist flies can oviposit and utilize a wide range of substrates (e.g., *D. melanogaster*, *D. simulans*, and *D. immigrans*), while specialists are typically restricted to a single substrate (Carton et al., 1991; Markow & O'Grady, 2008). For example, *D. phalerata* breeds in decaying stinkhorn mushrooms (Driessen et al., 1990), while *D. sechellia* is specialized on rotting morinda fruits that are toxic for other species in the melanogaster group (Markow & O'Grady, 2005). *L. heterotoma* predominantly attacks drosophilid larvae in fermenting fruits and sap fluxes, including *D. melanogaster* (Carton et al., 1991; Fleury et al., 2004; Janssen, 1989; Rizki et al., 1990), *D. simulans* (Carton et al., 1991; Janssen, 1989; Lynch et al., 2016; Papaj & Vet, 1990; Ris et al., 2004) and *D. suboscura* (Fleury et al., 2004; Janssen, 1989; Ris et al., 2004), and to a lesser extent *Drosophila* species breeding in decaying plant matter and fungi (e.g., *D. phalerata*; Janssen et al., 1988).

*Leptopilina heterotoma* can parasitize many different host species, but host suitability varies between species (Table S1). In a study by Janssen (1989), *D. kuntzei* was found to be the most suitable host for *L. heterotoma* with 89% of *L. heterotoma* offspring surviving, while *D. immigrans* was least suitable (2% wasp survival). In this study, *D. immigrans* was the only species (out of 9 species in total) where more hosts than *L. heterotoma* survived; hence *D. immigrans* was the least suitable host. *Drosophila immigrans* is indeed abundant in Europe but is rarely parasitized (Kraaijeveld & Godfray, 2009). The resistance of *D. immigrans* to parasitism was, however, suggested to result from its thick cuticle rather than the more typical immune response after parasitism (see below; Ideo et al., 2008; Kraaijeveld & Godfray, 2009; van Alphen & Janssen, 1982). In another study, development on *D. melanogaster* led to the highest percentage of surviving offspring (47%) compared to *D. suboscura* (30%), as well as *D. immigrans* and *D. suzukii* (<1%). Highest survival percentages (>85%) have been recorded on *D. melanogaster*, *D. hydei*, *D. kuntzei*, *D. pseudoobscura*, and *D. suboscura* (Table S1). Only very few *L. heterotoma* individuals survived when development occurred on *Zaprionus vittiger*, *D. suzukii* and *D. immigrans* (but see Hedlund et al., 1996) and no offspring survived when eggs were laid on *D. ananassae*, *D. biarmipes*, *D. paralutea* and *D. busckii* (Table S1). Survival on *D. melanogaster*, one of the preferred hosts of *L. heterotoma* (Carton et al., 1986, 1991; Fleury et al., 2004, 2009; Rouault, 1979) varies considerably between 26% and 93%, a difference that can be explained by several factors, including whether or not tested species shared an ecological history (hosts and wasps were collected from the same area at the same time), as well as genotype and geographic location (i.e., local adaptation; Fleury et al., 2004).

*Leptopilina heterotoma* and its drosophilid hosts are polyvoltine with multiple generations per year depending on habitat type, resource availability, and temperature (Fleury et al., 2009). Both wasps and hosts are thus present and/or active throughout most of the year, with the exception of winter (Fleury et al., 2009; Wertheim et al., 2006). *L. heterotoma* abundance is highest during summer, when higher temperatures lead to quicker development of both the wasps and their hosts. A field study by Godfray and Hardy (1990), for example, showed that wasps were abundant from June to September, with the highest number of individuals caught in June (i.e., up to 23 individuals caught per day), and a general decrease in numbers throughout July (13 per day) and August (9 per day). A more recent study by Knoll et al. (2017) in Switzerland also found that wasp abundance decreased from spring to autumn. Contrary to findings of Godfray and Hardy (1990) in the United Kingdom and of Mazzetto et al. (2016) in Italy where almost no individuals were found in September and October, respectively, Fleury et al. (2004) still found a high abundance of *L. heterotoma* in October in France. A study on the abundance of *Drosophila* and its parasitoids in Lyon, Valence, and Hyères (France) by Fleury et al. (2004) suggested that the seasonal abundance of *L. heterotoma* fluctuates in accordance with the abundance of the host *D. melanogaster*. Wasp abundance was found to depend on the respective location, with *L. heterotoma* being most abundant in Lyon where *D. melanogaster* also predominates. Remarkably low numbers of *L. heterotoma* have also been recorded, for example in the Southern sites in France (Valence and Hyères), resulting from a steep decrease in *D. melanogaster* numbers (Fleury et al., 2009). In Tunisia, *L. heterotoma* also nearly disappears when competitive interactions are high, with *D. simulans* and *D. buzzati* being the main hosts used (Carton et al., 1991). Abundance of *L. heterotoma* thus largely depends on geographic location, seasonality, local climatic conditions, host demography, and competition.

### 2.2 Host immunity

Encapsulation, a cellular immune response, is a process during which specialized haemocytes aggregate around the parasitoid egg and adhere to its surface to form a capsule. In the *melanogaster* host subgroup, these haemocytes are called lamellocytes, but within the larger Drosophilidae, several taxa evolved distinct types of haemocytes (e.g., pseudopodocytes in the *obscura* subgroup; see Wertheim, 2022 for a review). Melanization, which is part of the humoral immune response, entails the synthesis of melanin by lamellocytes that are encapsulating the parasitoid egg. This process occurs by the action of phenoloxidases that originate from haemocytes (Kacsoh & Schlenke, 2012; Nappi, 1975; Poyet et al., 2013). The combination of encapsulation by haemocytes and melanization prevents the parasitoid egg from hatching, eventually killing it (Streams, 1968).

Most *Drosophila* hosts fail to ignite an effective cellular (Nappi & Streams, 1969; Streams, 1968) and humoral immune response (Schlenke et al., 2007), and can thus not prevent the wasp embryo from developing (Poyet et al., 2013). Some host species, such as *D. suzukii* and *D. algonquin*, however, do have a strong immune response (Nappi, 1975; Poyet et al., 2013). Host resistance to parasitism likely depends on the level of circulating haemocytes, with more resistant species having higher haemocyte levels (Kacsoh & Schlenke, 2012; Poyet et al., 2013). Resistant hosts, such as *D. euronotus* and *D. algonquin* (Table S2), also possess immune pathways associated with the secretion of antimicrobial proteins and peptides, and other immune activities to inhibit egg/larval development inside the host (Nappi, 1970, 1975). An example is the induced changes in levels of a cell‐signaling molecule, nitric oxide, following parasitism (Carton et al., 2009). Even in species susceptible to parasitism by *L. heterotoma* or other parasitoids, laboratory experiments and observations with natural populations have shown that parasitism resistance is under strong selection and can increase in populations subjected to high parasitism risks (see Wertheim, 2022 for a review). Indeed, despite the high virulence of *L. heterotoma*, some hosts can acquire increased resistance through the evolution of novel genes, such as lectin‐24A in the *D. melanogaster* and *D. simulans* clade, which is implicated in the humoral response following parasitism by *L. boulardi* and *Asobara tabida* (Keebaugh & Schlenke, 2012). Increased parasitism resistance comes at cost, however, leading to trade‐offs, for example with host larval competitive ability or larval survival (Wertheim, 2022). Although the underlying immune response mechanisms of resistant hosts are now well understood, it has remained largely unclear how the host is able to recognize parasitoid eggs or larvae.

Among resistant host species, larvae of *D. suzukii*, known as the spotted wing *Drosophila*, are particularly efficient in killing *L. heterotoma* due to their high haemocyte load (Kacsoh & Schlenke, 2012; Poyet et al., 2013). *D. suzukii* mostly encapsulates developing wasps at the larval stage, rather than the egg stage (i.e., between 48 and 72 h post‐parasitism; Lacovone et al., 2018). *D. suzukii* originates from Asia and is a pest of economically important fruits, such as cherry, raspberry, blueberry, but also wild and ornamental plants (Kenis et al., 2016; Lee et al., 2015; Poyet et al., 2015). The fact that *D. suzukii* females lay their eggs on fresh fruits at a time very close to harvest makes the use of classic insecticides a risk for human health. Biocontrol agents are thus a preferable option (Rossi Stacconi et al., 2015). The efficient immune response of *D. suzukii* makes *L. heterotoma* almost unable to parasitize the larvae and is, therefore, not an ideal biocontrol agent against *D. suzukii* (Chabert et al., 2012; Girod, Rossignaud, et al., 2018; Kacsoh & Schlenke, 2012; Knoll et al., 2017; Poyet et al., 2013; Rossi Stacconi et al., 2017). Other parasitoids (e.g., *Trichopria drosophilae*, *Pachycrepoideus vindemmiae*), including those from the native region of *D. suzukii* in Asia (e.g., *Asobara japonica*, *Ganaspis brasiliensis*) seem able to parasitize and develop in this pest. These species can be investigated further for their potential use as biocontrol agents (Daane et al., 2016).

Immune responses can largely vary and depend both on biotic factors, e.g., age, developmental stage (Siva‐Jothy et al., 2005), and abiotic factors, e.g., temperature (Nappi & Silvers, 1984) or ethanol concentration (which is relevant because most *Drosophila* species develop on fermenting fruits; Lynch et al., 2017; Milan et al., 2012). An increased immune response can also be triggered by maternal effects, because *Drosophila* females produce offspring with increased lamellocyte production when oviposition occurs in the presence of *L. heterotoma* (Bozler et al., 2020). The *Drosophila*‐endosymbiont *Spiroplasma* (see Section 4) also plays a major role in *Drosophila* resistance against *L. heterotoma* (Corbin et al., 2021; Higareda Alvear et al., 2021; Paredes et al., 2016; Xie et al., 2011, 2014). By producing ribosome‐inactivating proteins, *Spiroplasma* seems to suppress development of the juvenile parasitoid by deactivating wasp ribosomes (Ballinger & Perlman, 2017). The protection conferred by *Spiroplasma* is temperature‐dependent, however, and is absent at 18°C (Corbin et al., 2021). The endosymbiont *Wolbachia* also increases *Drosophila* resistance to parasitism by *L. heterotoma*, albeit weak (Xie et al., 2014).

### 2.3 Parasitoid virulence

To overcome the host's immune response, some parasitoids inject venom during oviposition (Wertheim, 2022). In *L. heterotoma*, venom is known to affect host immunity leading to lysis of the host lymph gland (the organ responsible for the production of lamellocytes), thereby preventing the production of haemocytes (Ramroop et al., 2021). Venom fluids contain several components, including kinases, esterases and hydrolases (Heavner et al., 2013), but only few proteins have been accurately characterized up to now. Aspartylglucosaminidase (AGA) could be an important component of *L. heterotoma* venom (Colinet et al., 2013). This protein is abundant in *A. tabida* venom, where it is suspected to be involved in host paralysis during oviposition (Moreau et al., 2004). Haemocyte capsule formation around the parasitoid egg requires the glycosylation of proteins. AGA possesses deglycolsylation properties and may thus be involved in encapsulation prevention (Colinet et al., 2013). A recent study showed that a newly described protein, Lar (lymph gland apoptosis‐related protein), was abundant in *L. heterotoma* venom, promoting lysis of the host lymph gland (Huang et al., 2021). *L. heterotoma* venom also contains several other proteins, such as Elongation factor 1‐alpha (EF‐1α; Colinet et al., 2013), but its role in inhibiting the host's immune response has not yet been elucidated.

In many parasitoid species, including *L. heterotoma*, venom also includes virus‐like particles (Chiu et al., 2006; Colinet et al., 2013; Coulette et al., 2017; Goecks et al., 2013; Morales et al., 2005; Rizki et al., 1990). Virus‐like particles are produced in an accessory gland, also called the long gland or venom gland (Ferrarese et al., 2009; Rizki et al., 1990), and matured in a separate reservoir within the female wasp's reproductive system (Chiu et al., 2006; Morales et al., 2005). Virus‐like particles appear to be devoid of nucleic acids, but contain various proteins, among which the most abundant protein, p40, is located on the surface and spikes of mature particles (Chiu et al., 2006). The genes encoding virus‐like particles in *L. heterotoma* are embedded in the wasp genome (Huang et al., 2021; Wey et al., 2020) and could have originated from an ancestral virus (Di Giovanni et al., 2020). Other authors have, however, argued for a non‐viral origin of virus‐like particles and prefer the term mixed‐strategy extracellular vesicles (Heavner et al., 2013; Wey et al., 2020). Although the exact nature of the particles is still under debate, it is clear that virus‐like particles actively repress the host's immune response through several mechanisms. The particles are able to inhibit the functioning of lamellocyte adherence needed for encapsulation (Rizki et al., 1990; Rizki & Rizki, 1991) and to disrupt the generation of lamellocytes through lysing lymph glands (Chiu & Govind, 2002; Huang et al., 2021). Rizki and Rizki (1991) showed that virus‐like particles can enter lamellocytes and promote their lysis. The particles are also able to reduce the number of sessile haemocytes, another origin of lamellocytes (Anderl et al., 2016; Markus et al., 2009). The guaranteed immune suppression through virus‐like particles allows *L. heterotoma* to avoid encapsulation of its developing larvae by host lamellocytes and are thus essential for successful development.

## 3 HOST LOCATION, LEARNING, AND ADAPTIVE PATCH EXPLOITATION STRATEGIES

To produce offspring, female parasitoids need to be able to accurately locate and parasitize hosts. Successful parasitism results from a sequence of behaviors that include host habitat and patch location, host location within a patch, host acceptance, and host suitability (see Section 2; Godfray, 1994). Hosts are often distributed in isolated patches in the environment. To deal with such fragmented environments, parasitoid females need to divide their foraging efforts between different patches that can vary in host abundance during their lifetime, but also between generations (e.g., seasonal variation). Furthermore, in contrast to prey that become unavailable for competing predators, parasitized hosts remain on a patch and can subsequently be encountered by con or hetero‐specific female competitors. Most parasitoid females can discriminate hosts already parasitized by a conspecific, but discrimination of hosts parasitized by hetero‐specifics seems to be less common in parasitoids (Ardeh et al., 2005; Strien‐van Liempt & van Alphen, 1981). When encountering a parasitized host, the female can either reject it and continue to look for unparasitized hosts, or decide to lay an egg, a behavior known as superparasitism. While superparasitism is restricted to interactions with conspecifics, acceptance of a host parasitized by a hetero‐specific is referred to as multiparasitism. Superparasitism and multiparasitism, therefore, represents a combination of extrinsic (i.e., between females for access to hosts) and intrinsic competition (i.e., among parasitoid larvae within a host). Superparasitism comes at a risk though, because in solitary parasitoids only one adult can emerge from a single host, and the second egg generally has the lowest chance of survival (Bakker et al., 1985).

Since the 70's, the behavioral ecology of the amber wasp *L. heterotoma* has been extensively studied, mostly in the context of optimal foraging theory. This theory states that the time allocated and choices made while foraging for a resource are shaped by natural selection, maximizing fitness (Charnov, 1976). Research using *L. heterotoma* as a model revealed the importance of associative learning in patch and host selection in parasitoids. Due to its risky nature, superparasitism was long thought to be detrimental to fitness, but superparasitism can lead to fitness benefits for parasitoid females. Studies with *L. heterotoma* were instrumental to increasing our understanding of this phenomenon (Bakker et al., 1985). This section aims to present the sequence of *L. heterotoma* female behaviors, ranging from patch location to time allocated for foraging in a patch, illuminating the contribution *L. heterotoma* made to understanding how female parasitoid behaviors are shaped by natural selection.

### 3.1 Patch location

*Leptopilina heterotoma* females are attracted to the substrates on which *Drosophila* feed and oviposit (van Lenteren & Bakker, 1978; Vet & van Opzeeland, 1985), particularly by the presence and quantity of yeast and fermentation products (such as ethanol) resulting from substrate decay (Dicke et al., 1984; van Batenburg et al., 1983; van Lenteren & Bakker, 1978). These cues allow long‐distance detection of suitable host habitats (i.e., more than 1.5 m; Dicke et al., 1984), even when actual hosts are not present on the patch (Dicke et al., 1984; van Lenteren & Bakker, 1978). As host habitat odors do not necessarily imply the presence of hosts, these cues are not completely reliable. In addition to host habitat cues, *L. heterotoma* females can also eavesdrop to detect and locate host patches based on a host‐emitted pheromone: the *Drosophila* aggregation pheromone (Lof et al., 2013; Wertheim et al., 2003; Wiskerke et al., 1993). Aggregation pheromones (with cis‐vaccenyl acetate being the primary active compound; Bartelt et al., 1985) are deposited during oviposition by several *Drosophila* species to attract conspecific females (Bartelt et al., 1985; Wertheim et al., 2006). The aggregation pheromone is, therefore, a highly reliable cue indicating host presence for *L. heterotoma* females (Bartelt et al., 1985; Wertheim et al., 2006). Wertheim et al. (2003) showed that host aggregation pheromones indeed help *L. heterotoma* in finding host patches on smaller and larger spatial scales (i.e., a 40 cm wind tunnel and orchards, respectively). Attraction to host aggregation pheromones is further positively correlated with the concentration of yeast in the patch. Wasps were attracted by the aggregation pheromone of *D. melanogaster* when the yeast concentration was 2 g yeast/75 g of food medium, but wasps were not attracted when the yeast was less concentrated (1 g/75 g). To effectively locate host patches, *L. heterotoma* females thus use both habitat and host cues that in the natural environment may be amplified when combined, increasing their signal reliability for host finding.

### 3.2 Host location and choice within a patch

Once a female identifies and reaches a suitable patch, she starts to search for hosts by walking over the surface of the food substrate while rhythmically moving her antennae up and down and probing the substrate with her ovipositor (van Batenburg et al., 1983; van Lenteren, 1976; Vet & Bakker, 1985). She determines the exact location of the host when her ovipositor touches or pierces the host cuticle (van Lenteren, 1976; Vet & Bakker, 1985). Interestingly, the antennae seem of only little importance in these final steps of host location, because removal of the antennae does not prevent females from finding larvae, at least under laboratory conditions (van Lenteren, 1976). Once the female probes into a host, she can either reject it (i.e., withdraw her ovipositor in <6 s) or proceed to oviposit (lasting between 16 and 25 s; Haccou et al., 1991; van Lenteren, 1976; Varaldi et al., 2005). When a host's cuticle is pierced by the ovipositor, the host larva tries to escape by rotating itself and then moving away (van Lenteren et al., 1998). The ovipositor of *L. heterotoma* possesses a physical structure resembling a “clip” (see figure 1 in van Lenteren et al., 1998) that allows the wasp to constrain the larva and stop it from moving away while the female is injecting her venom (van Lenteren, 1976; van Lenteren et al., 1998). Following oviposition, the female then preens her ovipositor and genitalia. For a behavioral observer, this preening phase (in addition to oviposition duration) represents the second line of evidence that a female successfully laid an egg (Haccou et al., 1991; van Lenteren, 1976; Varaldi et al., 2005).

Once a female starts foraging in a patch, the presence of host aggregation pheromone is no longer of importance (Wertheim et al., 2003). To determine the presence and quantity of hosts feeding within a patch, females use host‐emitted kairomones (Dicke et al., 1985; van Alphen et al., 1984; Vet et al., 1993). Kairomones are semiochemicals that trigger a response from another species that are only beneficial to the receiver, not the emitter (i.e., the parasitoid and host larva, respectively; Grasswitz & Jones, 2002). Attraction to host kairomones is innate in *L. heterotoma*, because inexperienced females probe the substrate faster when host kairomones are present (Vet & Groenewold, 1990). When investigating a patch with host kairomones, *L. heterotoma* females intensify their searching behavior by spending more time on the area containing kairomones, and increasing the frequency of ovipositor probing (van der Hoeven & Vet, 1984). Host kairomones have not yet been identified chemically and could actually be compounds that originate from the adult flies or the larvae, such as cuticular hydrocarbons (CHCs) or feces (Dicke et al., 1985; van Alphen et al., 1984). Host‐produced kairomones are only detectable within a patch. This was substantiated by experiments performed on larger and smaller spatial scales: in a larger space (climate room), *L. heterotoma* did not visit patches containing hand‐deposited fly larvae (without aggregation pheromones, but with host kairomones; Dicke et al., 1984), while in a small space (5 cm Petri dishes), females were more attracted to patches on which larvae were feeding and crawling compared to host‐free patches (van Alphen et al., 1984). These studies highlight that while yeast odors and aggregation pheromone are of great importance for detecting patches from a distance of several meters (Dicke et al., 1984; Wertheim et al., 2003), host kairomones are critical for host location on a small spatial scale.

### 3.3 The role of associative learning in patch selection

Host patch selection by *L. heterotoma* females is not only influenced by chemical cues, but also by previous oviposition experiences, similar to other parasitoid species (Meiners, 2003; Sobhy et al., 2019). Through associative learning, females are more attracted to substrate odors on which they already had a successful oviposition experience (Simons et al., 1992; Vet et al., 1998; Vet & Schoonman, 1988; Vet & van Opzeeland, 1985). *L. heterotoma* females can use associative learning, for example, to differentiate between distinct substrates, e.g., apple‐yeast versus mushroom (Papaj et al., 1994; Papaj & Vet, 1990; Simons et al., 1992), and more similar substrates, e.g., pear versus apple (Vet et al., 1998). Females are not able, however, to differentiate between two different apple varieties (Vet et al., 1998). Associative learning also plays a role in finding host patches in the field. By doing experiments in a forest in the Netherlands, Papaj and Vet (1990) showed that experienced females tended to find artificial patches (containing apple‐yeast or mushroom substrates, without hosts) faster and more often than naive females. Experienced females were also more attracted to substrate types with which they had a previous parasitism experience. Overall, females seem capable of dynamically adjusting their search strategies in response to variability in environmental stimuli, including the availability and distribution of hosts in their environment (Vet et al., 1998).

Increased efficiency in patch finding with experience seems to result from a change in search activity: Vet and Papaj (1992) reused their protocol with apple‐yeast and mushroom substrates and tested how female experience affected searching behavior in terms of walking speed and direction. Experienced females changed direction less often and walked faster and straighter in the direction of an odor that they had previously experienced. Female preferences acquired through associative learning can, however, be reversed by an unsuccessful parasitism experience (i.e., not finding hosts) on an initially rewarding substrate, meaning that females are able to actively and rapidly adjust their search strategies depending on experience (Papaj et al., 1994). Associative learning also took place, but to a lesser extent, if the previous experience was not successful parasitism, but simply contact with host kairomones (Vet & Groenewold, 1990). Strong kairomone cues for host presence in the substrate thus also reinforces associative learning (Vet & Groenewold, 1990). Learning through processes other than association, like habituation or sensibilization to an environmental cue, do not lead to modifications of female preference (Vet & Groenewold, 1990). Altogether, laboratory and field experiments suggest that associative learning using cues based on host substrate and presence is adaptive when females face variable environments, playing an important role in microhabitat detection and selection under natural conditions.

### 3.4 The role of associative learning in parasitism success and superparasitism decisions

Learning is essential for the host location process, but also for parasitism success. Naive *L. heterotoma* females are less successful parasitizing hosts compared to experienced females, and a past oviposition experience decreases oviposition duration (Samson‐Boshuizen et al., 1974). *L. heterotoma* females are able to distinguish unparasitized hosts from hosts parasitized by conspecifics (Bakker et al., 1967, 1972; Hemerik et al., 2002; Visser, 1995) or themselves (Visser, 1993, 1995), also known as host discrimination. Females further have the ability to estimate the number of eggs already present in a host (Bakker et al., 1972, 1990; Hemerik et al., 2002; Visser, 1995). Like most other parasitoids (Ardeh et al., 2005), *L. heterotoma* females seem unable to discriminate hosts that are parasitized by other species, such as *A. tabida*, to avoid multiparasitism (Strien‐van Liempt & van Alphen, 1981; see Section 5 on competitive interactions). Host discrimination allows a female to estimate the quality of hosts within investigated patches, informing her about current oviposition conditions that can also have an effect on future oviposition opportunities (van Alphen & Visser, 1990). Early studies stated that females need a first experience of parasitism on already parasitized hosts to efficiently discriminate hosts (van Lenteren, 1972; van Lenteren & Bakker, 1975), but later work argued that hosts discrimination is innate in *L. heterotoma* (Henneman et al., 1995; van Alphen et al., 1987). In any case, host discrimination is due to chemosensory sensilla located on the distal part of the ovipositor (Ruschioni et al., 2015; van Lenteren, 1972; van Lenteren et al., 2007). When these sensilla come into contact with *D. melanogaster* hemolymph, the connected gustatory neurons produce action potentials (van Lenteren et al., 2007). These neurophysiological responses are dependent on the parasitism status of the host, as the number of action potentials differs significantly between unparasitized, singly, and doubly parasitized hosts (Ruschioni et al., 2015).

When two parasitoid eggs are deposited in the same host, the oldest individual within the host generally survives (Bakker et al., 1985; Eijsackers & Bakker, 1971), because it attacks and kills its competitor (Eijsackers & Bakker, 1971). In *L. heterotoma*, survival probability of the second larva is about 40% when laid shortly after the first larva (i.e., within 3 h), while the second larva never survives when laid after more than 24 h (Bakker et al., 1985; Visser, Luyckx, et al., 1992). Females mostly avoid superparasitism within the 3‐h window (Visser, Luyckx, et al., 1992). A potential explanation is that *L. heterotoma* marks its hosts during oviposition to prevent other females from superparasitizing. This mark does not, however, last more than 24 h. Similar to a previous experience with an unparasitized host (see above), an experience with a superparasitized host can modify subsequent oviposition decisions through learning (Henneman et al., 1995; van Alphen et al., 1987; Visser, van Alphen, et al., 1992). Visser et al. (1992) showed that oviposition experience on a patch containing only parasitized hosts leads to a higher rate of superparasitism on a new patch that contains both parasitized and unparasitized hosts after 24 h. When females forage alone, *L. heterotoma* does not superparasitize often (Varaldi et al., 2005), but the tendency to superparasitize increases when females investigate the patch simultaneously with other conspecifics (Bakker et al., 1985; Visser, 1995; Visser, Luyckx, et al., 1992; Visser, van Alphen, et al., 1992). Superparasitism rates further increase with the number of females simultaneously foraging in a patch (Visser et al., 1990). When a female is exposed to conspecifics before an experiment, but is subsequently left to forage on a patch alone, she also tends to superparasitize more than when she is kept alone (Visser, 1995).

Acceptance of previously parasitized hosts seemingly comes at a huge fitness cost for the female, but under extrinsic competitive pressure, superparasitism can be adaptive. When competition and the number of parasitized hosts in a patch are high, having at least some offspring that survive superparasitism is more advantageous than leaving the patch at the risk of not finding any hosts later on. Females are also more inclined to superparasitize hosts containing one of their own eggs (up to 30% of eggs were self‐superparasitized in Visser, 1995) when they are in competition with a conspecific female in the same patch (Visser, 1993, 1995; Visser et al., 1990). Here, self‐superparasitism could be adaptive, because it increases the fitness of the female by decreasing the probability that the host will be superparasitized by another competing female (van Alphen & Visser, 1990). By gathering information while searching for hosts, as well as learning from past oviposition experiences, *L. heterotoma* females can adaptively adjust their parasitism strategies in response to their environment. Research on *L. heterotoma* has emphasized that learning is of importance for parasitoids to choose patches that are more likely to contain hosts and to adjust superparasitism decisions, with a positive impact on fitness. Recent studies have, however, shown that learning in insects can come at a cost (de Bruijn et al., 2021; Mery & Kawecki, 2005), potentially leading to tradeoffs between learning capacities and life histories. Considering the extensive knowledge on learning in *L. heterotoma*, studying the cost of learning and potential trade‐offs can represent an interesting avenue for future research using this species.

### 3.5 Patch time allocation decisions: Learning from past experiences

A female can exploit multiple patches for oviposition during her lifetime; hence the time she spends within a patch can have a major effect on fitness (Hubbard & Cook, 1978). For example, if a new patch does not contain any hosts or only parasitized hosts, the female would have had a higher fitness if she had continued exploiting a previous, more suitable patch. Based on the marginal value theorem of optimal foraging (Charnov, 1976), patch allocation time depends on the fitness gain within a patch and the potential fitness gain expected on future patches available within the environment (Hubbard & Cook, 1978).

Similar to other parasitoids, patch time allocation in *L. heterotoma* is influenced by local conditions on the patch, including the number and quality of hosts encountered. For instance, the presence of kairomones, host encounters, and successful oviposition on a patch increase the time a female investigates that patch (Dicke et al., 1985; Haccou et al., 1991; van Alphen et al., 1984; van Lenteren & Bakker, 1978; Varaldi et al., 2005; Vet et al., 1993). Furthermore, females increase foraging efforts in new patches containing substrates on which they had a previous successful parasitism experience (Simons et al., 1992; Vet & Schoonman, 1988). Patch residence time further increases when superparasitism occurs, as it is adaptive for females to allocate more time to a patch with conspecifics to increase (self‐)superparasitism (Visser et al., 1990). In contrast, when the time between ovipositions increases (Haccou et al., 1991) or parasitized host encounters are getting more frequent (van Alphen & Vet, 1986; van Lenteren, 1991; Varaldi et al., 2005), females have a higher tendency to leave a patch. When females experience such poor conditions, they will spend more time finding hosts in a new, different type of substrate (Visser, van Alphen, et al., 1992).

Most optimal foraging models rely on oversimplified assumptions, such as a global knowledge of the organism's environment in terms of prey/host density, distance between patches, etc… Such assumptions are clearly unrealistic, leading to some criticism within the scientific community (Pierce & Ollason, 1987). These earlier studies were, however, necessary for new optimal foraging studies to build upon (King & Marshall, 2022). More recent optimal foraging models include the notion that foraging behaviors are dynamic and change within the lifetime of an individual (King & Marshall, 2022). For example, patch entering decisions and time allocated to a patch depend on the internal physiological state of females, including energetic reserves, age and mating status (Zhang et al., 2022), as well as climatic conditions (Roitberg et al., 1992), and learning. *L. heterotoma* would be a great model to test more recent optimal foraging models to further develop optimal foraging theory.

### 3.6 Influence of seasonal factors on parasitism strategies and fitness

Seasonal changes can have major effects on insect behavior and fitness (Abram et al., 2017), including parasitism strategies. In *L. heterotoma*, females are indeed known to adjust parasitism strategies in preparation for winter (Roitberg et al., 1992). For example, changes in photoperiod modify host patch exploitation, as wasps reared under autumn‐like light conditions (16 L:8D, 22°C) investigate host patches longer and superparasitize more often compared to wasps reared under summer conditions (12 L:12D, 22°C). These behavioral adjustments could be due to the shorter life expectancy of autumn females, leading to a riskier oviposition strategy (Roitberg et al., 1992), following the relative fitness rule. This rule states that when facing deleterious environmental conditions, parasitoids should adopt a riskier strategy maximizing the chances that their genes will be represented in the next generation (Giraldeau & Boivin, 2008). Furthermore, *L. heterotoma* survival in multi‐parasitized *D. melanogaster* larvae is lower at a cold (15°C) compared to a higher temperature (25°C; Strien‐van Liempt, 1983). Host choice in terms of host species can also affect thermal stress resistance. For example, survival and female fecundity at low (14–18°C) or high (26°C) temperatures are lower when wasps are developing on less suitable hosts (see Section 2 on host suitability), such as *D. simulans* or *D. subobscura* (compared to *D. melanogaster*; Fleury et al., 2004; Ris et al., 2004).

## 4 THE EVOLUTION OF FAT ACCUMULATION AND CONSEQUENCES FOR LIFE HISTORIES

The ability to accumulate fat is a highly conserved metabolic process across all domains of life (Birsoy et al., 2013; Wältermann & Steinbüchel, 2005). During periods of food abundance, animals, including insects, use dietary nutrients to meet acute energetic demands, while excess sugars and other carbohydrates are converted to fat for long‐term energy storage. Fat is thus a critical source of energy for insects to invest in survival and reproduction, particularly when faced with harsh environmental conditions (Arrese & Soulages, 2010; Hahn & Denlinger, 2011; Sinclair & Marshall, 2018). Fat is further important for other traits, such as locomotor activity, desiccation resistance, and as a macronutrient in eggs (Arrese & Soulages, 2010; Muller et al., 2017). Body size (a proxy for fat reserves, because size and fat content are generally correlated in arthropods; Enriquez et al., 2022; Lease & Wolf, 2011), longevity, and reproductive output are common life history traits for assessing fitness in insects (Roff, 2001). Trade‐offs between longevity and dispersal (e.g., flight), as well as longevity and reproduction have been well documented (Blacher et al., 2017; Chang et al., 2021), where fat allocation underpins both trade‐offs. The tight relationship between fat reserves and fitness thus makes the study of fat accumulation of importance for both ecological and evolutionary studies.

### 4.1 Fat accumulation

Despite the critical importance of fat reserves, it was only in the early 1990s that adult parasitoids were found unable to accumulate fat including the amber wasp *L. heterotoma* (Eijs et al., 1998; Ellers, 1996). Using laboratory‐reared individuals, Eijs et al. (1998) were the first to test the effect of multiple food resources (natural, non‐breeding, and artificial substrates) on fat accumulation of adult *L. heterotoma*. Fat content of *L. heterotoma* was highest at emergence and declined despite continuous feeding on honey, and irrespective of the *Drosophila* host used for development. Together with data on other parasitoid species, this lack of fat accumulation was hypothesized to result from the parasitoid lifestyle (Visser & Ellers, 2008). Only parasitoid insects were thus expected to lack fat accumulation, because sufficient fat for allocation into life history traits could be carried over from the host during development. A comparative study with more than 90 insect species then showed that the ability for fat accumulation was indeed lost during the course of evolution, but only in parasitoid lineages (including flies, a beetle, as well as parasitic hymenopterans) and not in other insects (Visser et al., 2010). For *L. heterotoma*, the results of Visser et al. (2010) differed from those obtained by Eijs et al. (1998), because in the former fat content significantly increased during life, showing that fat had been accumulated. In at least two other parasitoid clades the fat accumulation phenotype seemed to have re‐appeared in generalists, suggesting that adult fat accumulation could have re‐evolved in wasps with a large host range, including *L. heterotoma* (see Section 2 and Table S1). A repetitive loss and regain of fat accumulation suggests a modification of gene expression, rather than genetic changes in coding sequences of fat synthesis and accumulation genes (Visser et al., 2012). In addition, Moiroux et al. (2010) found that fat accumulation ability differed between geographically distinct *L. boulardi* populations (reared on the same host species), suggesting local adaptation depending on the environmental settings.

Following the contradictory findings in *L. heterotoma* and the intra‐specific differences observed in *L. boulardi*, Visser et al. (2018) conducted a large‐scale study on the ability of 19 field‐caught *Leptopilina* populations (belonging to different species) to accumulate fat in 2016. Thirteen out of 19 populations were *L. heterotoma* and these populations were compared to earlier work on 9 geographically distinct *L. heterotoma* populations collected from the field in 2013. For the populations collected in 2013, similar results were obtained as in Moiroux et al. (2010): some populations lacked fat accumulation, while other populations significantly increased fat content during life. In contrast, the populations obtained in 2016 (as well as the other species tested) all lacked fat accumulation. That puzzling finding resulted from differences between the *Drosophila* host strains used. The *D. melanogaster* strain used for the 2013 populations was collected from the field and was much leaner compared to the laboratory‐reared strain used for testing the 2016 populations. This became evident when fat content of recently emerged *L. heterotoma* females were compared between years: females contained almost twice as much fat in 2016 compared to 2013, explaining why no fat accumulation was observed in any of the 2016 *L. heterotoma* populations or the other species.

Variation in fat accumulation between *L. heterotoma* populations was hypothesized to be the result of phenotypic plasticity (i.e., fat accumulation depends on host fat content). A recent study with *L. heterotoma* indeed confirmed that fatty acid synthesis and fat accumulation depend on host fat content (that can easily be manipulated in the laboratory; Enriquez et al., 2022). Fatty acid synthesis and fat accumulation mainly occurred when the wasps developed on lean hosts, but was shut off on fat hosts (Visser et al., 2021). Reaction norms for fatty acid synthesis also differed considerably between *L. heterotoma* populations, suggesting that fat synthesis regulation can occur rapidly when host fat content varies and is dependent on the wasp's genotype. *L. heterotoma* thus represents an interesting example of a parasitoid that shows adaptive phenotypic plasticity in a key physiological trait.

Fat synthesis, accumulation and plasticity therein in *L. heterotoma* is currently the core theme of our own research and there are many exciting prospects for further research on this topic (Visser et al., 2022). For example, we still need to better understand how plasticity of fat synthesis and accumulation affects life histories and fitness (see the subsection below). We can further use field‐caught populations to elucidate how phenotypic plasticity itself evolves in different natural environments. So far, explicit tests for plasticity of fat synthesis and accumulation have only been done with *L. heterotoma* (using genetically similar individuals). To determine if plasticity of fat synthesis and accumulation evolved also in other parasitoids, many more parasitoid species now need to be tested (Visser et al., 2022). We can also now start digging into the genomics and transcriptomics of plastic fat synthesis in *L. heterotoma* to understand the mechanisms at play in generating distinct fat accumulation phenotypes.

### 4.2 Life histories

Evidence for the close link between fat reserves, critical as a long‐term energy source, and key life history traits in parasitoids comes largely from earlier work on the *Drosophila*‐parasitizing braconid wasp *A. tabida*. The importance of fat reserves for *A. tabida* reproductive functions was demonstrated by the positive correlation between the quantity of fat and female egg load (i.e., fatter females have more eggs in their ovarioles at emergence; Ellers, 1996; Le Lann et al., 2014). Moreover, a higher fat content leads to higher adult survival (Ellers, 1996). Fat reserves also fuel *A. tabida* locomotion, as fat reserves decreased with increasing dispersal distance (Ellers et al., 1998). Similar to most other parasitoids, *A. tabida* does not accumulate fat (Ellers, 1996), limiting the amount of fat reserves available for fitness functions. Fat content of *A. tabida* indeed decreases quickly during the first week of life, when many eggs are laid (Ellers, 1996). During this time, fat reserves are thus mostly allocated towards reproduction, leading to trade‐offs with other life history traits (Ellers, 1996). Although more studies on *L. heterotoma* are now appearing, particularly concerning fat synthesis and accumulation (see above), very little is known about the link between fat content, life histories, and trade‐offs. Preliminary work using *L. heterotoma* confirms the major importance of fat reserves, at least for survival, because fat content at emergence determines longevity under starvation for males (i.e., fatter males have a longer lifespan) (B. Visser, unpublished data, Table 2).

**TABLE 2:** Life history trait measurements of *L. heterotoma* (B. Visser, unpublished data)

Population	Host diet	Male longevity under starvation (days)		Offspring number		Offspring sex ratio (number of males/total offspring)	
		Mean	*1 SE*	Mean	*SE*	Mean	*1 SE*
Lh8, Japan	Lean	5.95	0.22	73.67	10.05	1	0
	Control	8.21	0.62	33.88	4.43	0.47	0.12
	Fat	10	0.32	15.33	3.96	0.47	0.16
Lh9, UK	Lean	5.47	0.37	36.88	4.63	0.41	0.13
	Control	7.44	0.34	41	6.36	0.53	0.14
	Fat	9.45	0.21	38.25	7.05	0.29	0.07
Lh10, UK	Lean	5.58	0.43	39.63	4.36	0.70	0.14
	Control	6.86	0.43	37.44	4.60	0.37	0.09
	Fat	8.60	0.76	53.50	8.98	0.47	0.16
Lh13, Belgium	Lean	6.33	0.23	73.29	3.99	0.46	0.14
	Control	8.14	0.28	54.50	8.64	0.42	0.09
	Fat	9.13	0.29	56.71	7.97	0.29	0.03

*Note*: For each trait the mean (±1 *SE*) is provided. Data were obtained from wasps reared at 23°C with females ovipositing on lean, control, and fat *D. melanogaster* hosts (obtained as in Enriquez et al., 2022; Visser et al., 2021). Longevity under starvation was determined for males that developed on lean or fat hosts.

Offspring sex ratios of parasitoid wasps have been of particular interest in the context of local mate competition theory (see Section 7; Godfray & Cook, 1997; Hamilton, 1967), but host quality can also affect sex allocation patterns (Charnov, 1979, 1982; Clark, 1978; Godfray, 1994; Hardy, 1994; Visser et al., 2014). Charnov theorized that sex allocation of parasitoid females depends on host quality (typically measured as host size) when host quality affects the fitness of sons and daughters differently (Charnov, 1979; Charnov et al., 1981). Charnov's model assumes that host body size (and associated fat content, see above) is a key determinant of both female and male fitness. The relationship between size and fitness is even more important for females, as they benefit more from being large compared to males (i.e., a higher reproductive success and fecundity are typically proportional to host size). Males are then laid in smaller hosts, while females are laid in larger hosts, optimizing host exploitation. For several parasitoid species, the proportion of males was indeed shown to decrease with increasing host size (Charnov, 1982; Godfray, 1994; King, 1993). In *L. heterotoma*, sex allocation also seems to be dependent on host quality: sex ratios are generally male‐biased when females lay eggs on lean hosts, and female‐biased on fat hosts (B. Visser, unpublished data; Table 2). Variation in *L. heterotoma* offspring sex ratios also appears to be dependent on the wasp population (Table 2). It remains unclear, however, if and how parasitoid females can estimate host size, which can vary largely in time and space.

Clark (1978) proposed that local resource competition can also affect sex allocation. If resources are locally limited, parasitoid females may be forced to compete with each other females for access to resources (Visser et al., 2014). Under such circumstances, mothers limit competition among daughters and allocate more resources by producing sons that can disperse (male‐biased sex ratio). As a result of increasing temperatures, *L. boulardi*, a major competitor of *L. heterotoma*, is migrating towards more northern parts of Europe, replacing *L. heterotoma*. The presence of *L. boulardi* results in higher mortality and lower host availability for *L. heterotoma* (Fleury et al., 2004). To cope with increased competition, higher fecundity and investment in mobility (to be able to find more suitable hosts), coupled with a shorter life span (that is traded‐off) are expected based on the balanced mortality assumptions of Price (1974). However, no clear distinction in life history traits between *L. heterotoma* populations, with or without *L. boulardi*, was found (Vayssade et al., 2012; Vuarin et al., 2012). Moreover, host‐ (e.g., age, sex, or species) or wasp‐ (e.g., species, genotype, maternal size, age, diet, or microhabitat) related traits need to be considered in future studies on parasitic wasp sex ratios, including *L. heterotoma* (Chabora et al., 1979; King, 1987).

Endosymbionts can have a major impact on their host, including life histories (see Section 7 on cytoplasmic incompatibility). For *L. heterotoma* attacking *Spiroplasma*‐infected and uninfected *Drosophila*, no differences were, however, found in the number of eggs laid (Paredes et al., 2016; Xie et al., 2010, 2014). A recent study on *Spiroplasma* showed that this endosymbiont actually subverts specific host lipids and its proliferation is limited by the availability of host hemolymph‐lipids (Herren et al., 2014). *Spiroplasma* and wasp thus seem to compete for *Drosophila* host resources, a pattern already reported for *L. boulardi* (Paredes et al., 2016). The presence of *Spiroplasma* in some *Drosophila* hosts can thus have major consequences for lipid availability during development of *L. heterotoma*, a factor known to affect fat acumulation in adults.

Availability and quality of resources, as well as abiotic factors, such as temperature, are fluctuating at different temporal scales in the environment (between years, seasons, days,…). Temperature is known to have a major effect on female parasitoid behavioral decisions (i.e., foraging, host choice; Amat et al., 2006; Moiroux et al., 2015) that can affect offspring nutrient acquisition during development and consequently fat accumulation and fitness. *L. heterotoma* occurrence is widespread, which is typically associated with a high tolerance to a wide range of temperatures (Addo‐Bediako et al., 2000; Sunday et al., 2012). Life histories in *L. heterotoma* seem to be optimal between 20 and 23°C. Indeed, survival of developing *L. heterotoma* (Ris et al., 2004; Rossi Stacconi et al., 2017), fecundity of females (Fleury et al., 2004; Le Lann et al., 2014; Ris et al., 2004), and parasitism success (Rossi Stacconi et al., 2017) decrease at lower (14–18°C) or higher (25–35°C) temperatures. Temperature further has a significant effect on resource‐use strategies of *L. heterotoma*: females reared at 20°C accumulated a significant amount of fat reserves, whereas individuals at 23°C did not accumulate fat (Le Lann et al., 2014). More studies are now needed to fully appreciate how temperature, and fluctuations therein, affect resource acquisition, use (i.e., fat accumulation phenotypes), as well as life histories and trade‐offs in *L. heterotoma*.

## 5 PARASITOID SPECIES COEXISTENCE

There are currently more than 2000 recorded species within the host fly subfamily Drosophilinae (O'Grady & DeSalle, 2018). Within the genus *Drosophila*, there have been several major adaptive radiations, and some lineages have high diversification rates related to resource‐use (Markow & O'Grady, 2008). Although the number of parasitoids known to attack *Drosophila* species are underestimated (Lue et al., 2021), there is already high intra‐ and interspecific competition for hosts within the guild of parasitoids associated with *Drosophila*. In this section, we introduce the guild of *Drosophila* parasitoids and discuss species abundances in Europe and Asia. We further describe how competition for host resources can lead to potential speciation, and how spatial and temporal resource partitioning allows species coexistence.

**FIGURE 4:**
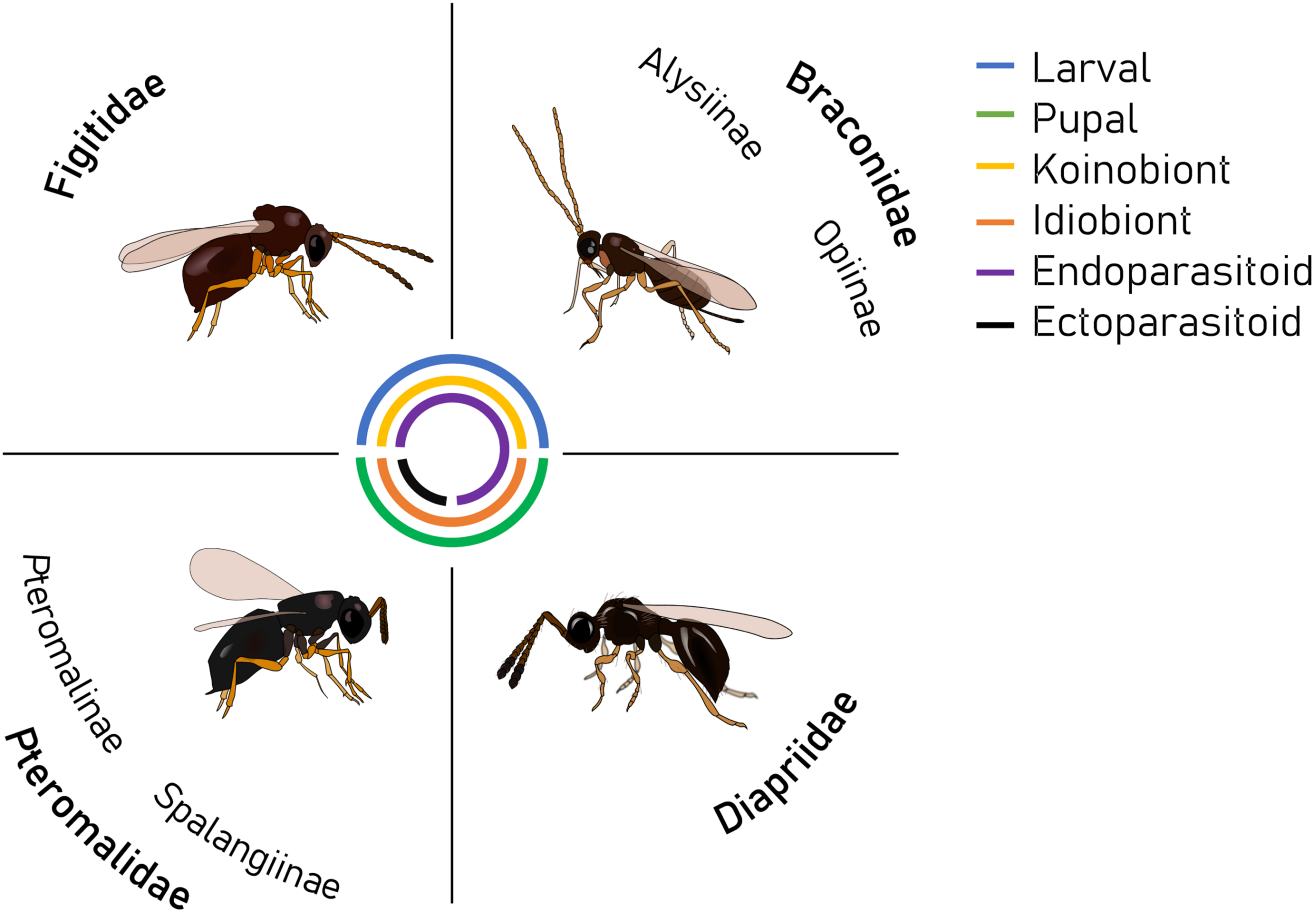
Lifestyle characteristics of the four main wasp families parasitizing *Drosophila*. Each family is visually represented by a common species: Figitidae—*Leptopilina heterotoma*, Braconidae—*Asobara tabida*, Pteromalidae—*Pachycrepoideus vindemmiae*, Diapriidae—*Trichopria drosophilae*. Eggs of ectoparasitoids are laid on the outside of the host, whereas those of endoparasitoids are laid inside the host. Endoparasitoid larvae may, however, develop some time outside the host body, depending on the species (see Figure 3 for *L. heterotoma* where this occurs; Harvey & Strand, 2002).

The amber wasp *L. heterotoma* belongs to a large guild of parasitoids attacking *Drosophila* species, with a current count of 108 species belonging to 20 genera (Carton et al., 1986; Lue et al., 2021; Table S2). The use of *Drosophila* hosts evolved independently in the superfamilies Ichneumonoidea, Cynipoidea, Chalcidoidea, and Diaprioidea. Hosts are attacked either during the larval stage (e.g., *Leptopilina*, *Ganaspis*, *Asobara*, *Opius*) or the pupal stage (e.g., *Pachycrepoideus*, *Spalangia*, *Trichopria*; Carton et al., 1986). All larval parasitoids of *Drosophila* are endoparasitoids, while the pupal parasitoids are either ectoparasitoids (i.e., Pteromalidae) or endoparasitoids (i.e., Diapriidae; Figure 4; Carton et al., 1986). *P. vindemmiae* and *Spalangia* sp. were further found as secondary parasitoids, also called hyperparasitoids, on primary hymenopteran (e.g., *Leptopilina* and *Asobara* species) or dipteran hosts (Gibson, 2009; van Alphen & Thunnissen, 1982). In terms of developmental strategies, all braconids attacking *Drosophila* are koinobionts (i.e., allowing host growth after parasitism), while species in the subfamilies Pteromalinae and Spalangiinae are idiobionts (arresting host development). The guild of parasitoid species associated with *Drosophila* thus shows great diversity in host exploitation strategies.

In Europe, the larval endoparasitoids *L. heterotoma*, *L. boulardi* and *A. tabida* are common (Fleury et al., 2009; Knoll et al., 2017; Mazzetto et al., 2016), sharing different host species, such as *D. melanogaster*, *D. simulans*, and *D. subobscura* (Fleury et al., 2004, 2009; Kraaijeveld & Godfray, 1999). *D. phalerata* is the most abundant fungal‐feeding host and is parasitized mainly by *L. clavipes* (Driessen et al., 1990). Among the pupal parasitoids, *P. vindemmiae*, *T. drosophilae*, and the genus *Spalangia*, are the most common in Europe (Delpuech & Allemand, 2011; Fleury et al., 2009; Kremmer et al., 2017). Data on the occurrence of *Drosophila* parasitoids and their hosts are relatively scarce outside Europe and Asia (but see Abram et al., 2022; Lue et al., 2018 for data in North America, and Jeffs et al., 2021 for data in Oceania). *P. vindemmiae* and *T. drosophilae*, which are cosmopolitan, are the main pupal parasitoids in Asia (Daane et al., 2016; Giorgini et al., 2019; Mitsui et al., 2007). In Japan, the most common drosophilids feeding on fruits in temperate regions are the native *D. lutescens*, *D. suzukii*, and the exotic *D. simulans* and *D. immigrans* (Kimura et al., 1994; Mitsui et al., 2007; Mitsui & Kimura, 2010). Parasitoids attacking these species are *A. japonica*, which has a remarkably large host range, and *G. brasiliensis* (currently considered as a cryptic species; Kimura & Mitsui, 2020; Mitsui et al., 2007; Mitsui & Kimura, 2010). The same species are found in South Korea (Daane et al., 2016), while in China *G. brasiliensis*, *L. japonica* and *A. mesocauda* are the most common parasitoids (Giorgini et al., 2019; Girod, Borowiec, et al., 2018). The *G. brasiliensis* lineage that specializes on *D. suzukii* could represent a suitable biocontrol agent against this pest (Nomano et al., 2017), once the species within this complex are formally described (Seehausen et al., 2020).

### 5.1 Cryptic species

**FIGURE 5:**
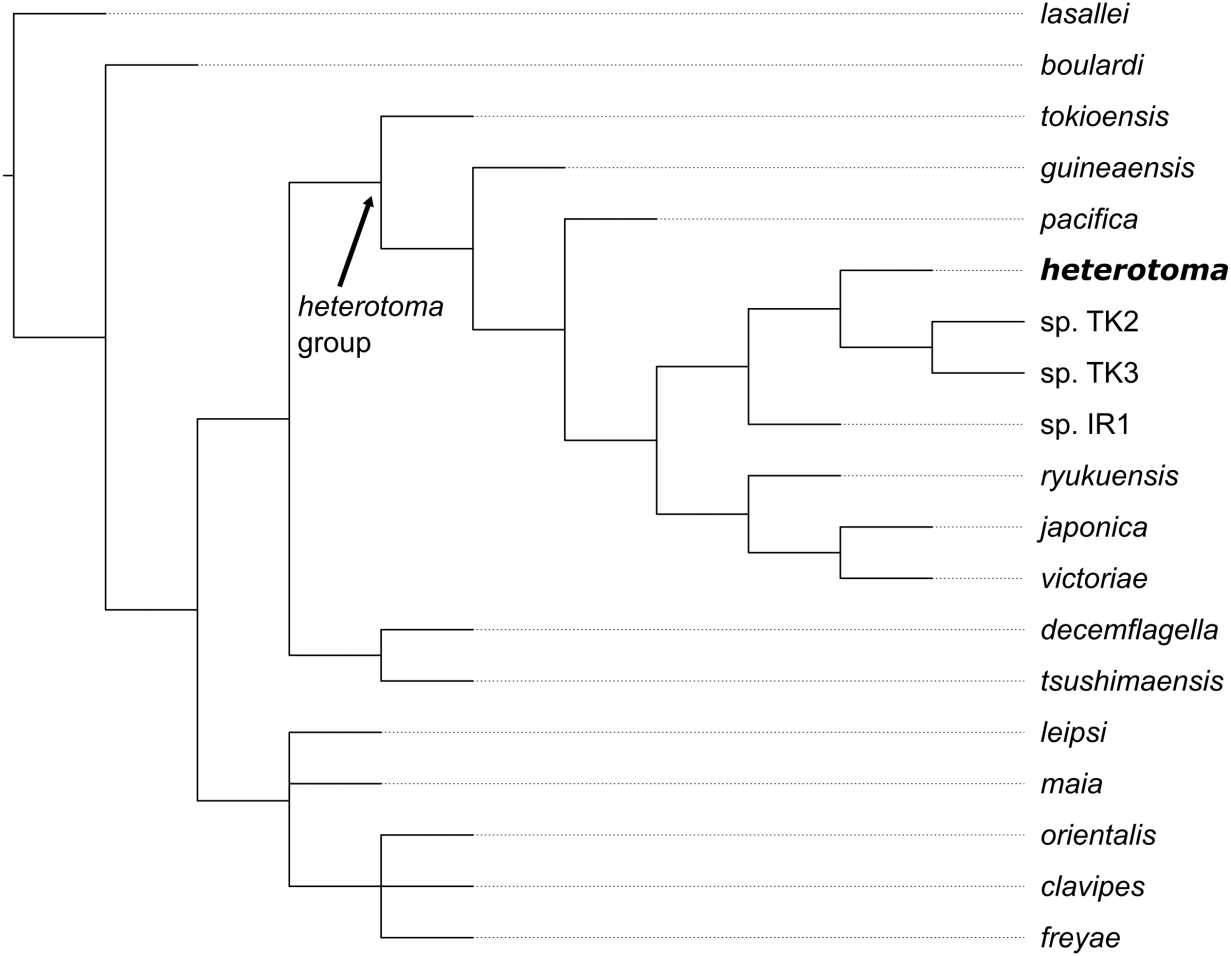
Phylogeny of the genus *Leptopilina*, redrawn from Novkovic et al. (2011) and Buffington et al. (2020).

When resources, such as hosts, are limited competition between species intensifies. An outcome of intense competition is competitive exclusion, where one of the competing species ultimately goes extinct (Losos, 2000). Alternatively, natural selection can favor phenotypes within a population that avoid resource competition. Populations can thus diverge in resource use, lowering competition and allowing species coexistence, potentially leading to speciation. *L. heterotoma* belongs to a species‐rich genus, containing more than 30 species, that is divided into several groups, including a *L. heterotoma* group (Figure 5). Two species within this group have a broad distribution (*L. heterotoma*, *L. victoriae*), while the other species are restricted to Asia (*L. pacifica*, *L. ryukyuensis*, *L. japonica*, *L. tokioensis*) or Africa (*L. guineaensis*). Some of these species have only recently been described and their biology still remains largely unknown (Novkovic et al., 2011; Wachi et al., 2015). *L. heterotoma* is distributed across the temperate regions of Europe, Asia, North America and Oceania. It has been observed up to Sendai in Japan, although the records furthest South (Tokyo) were recently proposed to be cryptic species (based on sequencing of neutral mitochondrial and nuclear markers; Kimura & Mitsui, 2020; Novkovic et al., 2011).

Considering that hymenopteran parasitoids belong to one of the most diverse insect orders (Forbes et al., 2018), it is not surprising that an increasing number of cryptic parasitoid species are being discovered (Gokhman, 2018). *L. heterotoma* from Sendai and Sapporo appear genetically most similar to European strains, two potential cryptic species were identified in Tokyo, and the genetically most divergent species was caught on the islands Iriomote and Amami‐oshima (Novkovic et al., 2011; Visser et al., 2018; Figure 5). The three cryptic species indeed appear to have shifted host use, with one of the Tokyo species parasitizing mainly *D. bizonata* breeding on mushrooms, the other Tokyo strain parasitizing *Scaptodrosophila coracina* breeding on fruits, and the island species mainly parasitizing *Lissocephala* species that breed on figs. It is still unclear whether the strains identified can still interbreed, but these potentially cryptic species offer interesting opportunities to study speciation in action (Struck et al., 2018).

### 5.2 Niche partitioning

Over shorter time scales, competition for hosts can be reduced through temporal or spatial niche partitioning (Germain et al., 2018; Harvey et al., 2014; Kronfeld‐Schor & Dayan, 2003). Due to its broad distribution across the world, *L. heterotoma* interacts with and can face severe competition from other wasp species, mainly those attacking frugivorous *Drosophila*, such as its congener *L. boulardi* and the braconid *A. tabida*. Indeed, no clear spatial niche differentiation seems to be apparent for these three species (Fleury et al., 2009). A study performed in the UK showed that *A. tabida* and *L. heterotoma* are abundant and co‐occur from May to September (Godfray & Hardy, 1990), while in the Southeast of France *L. heterotoma* and *L. boulardi* dominate with relatively few *A. tabida* individuals emerging from April to September (Fleury et al., 2009). In Tunisia, *L. heterotoma* faces intense competition from *L. boulardi*, which is probably causing *L. heterotoma*'s competitive exclusion during most of the season (Carton et al., 1991). Abundance of competing parasitoids in the Southeast of France seems to follow that of the different host species (Fleury et al., 2009). The geographic range of *L. boulardi* is restricted to the Mediterranean, where the host *D. simulans* dominates, while in the North *L. heterotoma* thrives developing on *D. melanogaster*. *L. heterotoma* abundance also reaches only a few percent when *L. boulardi* is present. Under such intense competition, *L. heterotoma* seems to persist as a result of phenological differences between parasitoid species, with *L. heterotoma* being present and most abundant only very early and late in the season. This is possible, because unlike *L. boulardi*, *L. heterotoma* does not diapause in winter (Carton et al., 1991; Kimura, 2019).

*Leptopilina heterotoma* seems to have a competitive advantage when *L. boulardi* females are infected by a virus, the *L. boulardi* filamentous virus (LbFV) that increases the rate of superparasitism (Section 3). As a consequence, fewer offspring of infected *L. boulardi* females reach adulthood allowing *L. heterotoma* to predominate, at least in laboratory experiments (Patot et al., 2012). In the field, 55%–95% of *L. boulardi* females may be infected with LbFV, depending on the location, with infection increasing towards the South (and being absent in the North). Considering the drastic effect of LbFV infection on *L. boulardi*'s parasitism strategy, it can be expected that competitiveness is lowered in infected *L. boulardi* also in natural populations, but this remains to be tested.

On even smaller spatial scales, *L. heterotoma* can avoid competition using chemical cues to select a preferred microhabitat for oviposition. For example, Vet and van Opzeeland (1985) showed that *L. heterotoma* prefers substrates that are in a later stage of decay, compared to *A. tabida* that prefers substrates at an early stage of decay (Vet et al., 1984). These findings confirmed anecdotal field observations where *A. tabida* appeared near substrates about the same time as the hosts, while *L. heterotoma* appeared only later (Vet & van Opzeeland, 1985). Due to differences in the temporality of parasitism between *A. tabida* and *L. heterotoma*, where hosts parasitized by *A. tabida* are likely already at the pupal stage, multiparasitism and direct competition can be avoided. Furthermore, circadian rhythms leading to temporal segregation can also contribute to coexistence between the three main competing *Drosophila* parasitoids. In a study by Fleury, Allemand, et al. (2000), the authors compared the circadian rhythms of *L. boulardi*, *A. tabida* and *L. heterotoma*, revealing that within a single day, *L. heterotoma* and *A. tabida* are active and ovipositing earlier than *L. boulardi*. Both *L. heterotoma* and *A. tabida* can thereby avoid competition with *L. boulardi*, the strongest intrinsic competitor (see the next subsection; Allemand et al., 1999; Carton et al., 1991).

Once competitors do arrive at the same patch, competition can still be avoided: Janssen et al. (1995) showed that when *L. heterotoma* interacts with its congener *L. clavipes* on decaying stinkhorn patches, *L. heterotoma* avoids patches where *L. clavipes* is present. The same avoidance strategy was found when *L. heterotoma* would encounter patches with *L. boulardi*. Weiss et al. (2013) indeed showed that *L. heterotoma* females avoided host patches that were already occupied or exploited by both conspecific and heterospecific female wasps, as well as wasp extracts. *L. heterotoma* females thus use different environmental factors to avoid competition on larger and smaller spatial and temporal scales.

### 5.3 Intrinsic competition

When competitors cannot be avoided and egg laying occurs in the same patch, *L. boulardi* outcompetes *L. heterotoma*. When both species were allowed to lay eggs at the same time with access to the same host (*D. simulans*), *L. heterotoma* parasitism rate was reduced from 50% (parasitizing alone) to 30% (together with *L. boulardi*; Carton et al., 1991). Furthermore, *L. heterotoma* developmental success was also reduced, from 51% to 37%. Similar patterns were found by Fleury et al. (2009) using two host species (*D. melanogaster*, *D. simulans*), although genotypes originating from more Southern populations in France were better at competing with *L. boulardi* (~30% *L. heterotoma* emergence) compared to Northern populations (~10% *L. heterotoma* emergence). This suggests that there is local adaptation for increased competitive ability in populations where *L. heterotoma* and *L. boulardi* co‐occur.

Once a host contains more than one developing parasitoid, intense intrinsic competition is unavoidable, because only one parasitoid can utilize and survive on one host. In experiments where *A. tabida* and *L. heterotoma* were laid in the same host (*D. melanogaster*), generally one of the competitors is eliminated through physical attack by the first hatched larva (Strien‐van Liempt, 1983). Which species survives depends on several factors, including the time interval between oviposition, temperature, and multiparasitism. Studying coexistence and competition between *Drosophila* parasitoids is now particularly relevant in the context of climate warming, as *L. boulardi* is migrating northwards, leading to population (and potentially genetic) differentiation in thermal reaction norms of life histories in marginal populations (Delava et al., 2022). Future studies on the consequences of the recent range expansion of *L. boulardi* on competitive interactions can help to better understand and predict the effects of climate change.

## 6 CHEMICAL COMMUNICATION AND SEMIOCHEMICAL PARSIMONY

Chemical communication probably constitutes the oldest and most widespread form of communication, occurring in all domains of life (Wyatt, 2014). Although several hundred sex pheromone components (i.e., molecules involved in mating behavior or related processes between individuals of the same species; Wyatt, 2010) have been identified (El‐Sayed, 2022), the origin and evolution of sex pheromones are still not well understood for most animals. Most insects produce sex pheromones to stimulate mating behavior through sexual attraction. The release of sex pheromones may be related either to the attraction of the opposite sex, generally via highly volatile compounds released by females to attract males over long distances, or as a part of male courtship behavior at closer range, generally via low volatile compounds (Ayasse et al., 2001; Kohl et al., 2015; Renou, 2014).

In the amber wasp *L. heterotoma*, iridoids play a key role in the mate‐finding process (i.e., in the attraction of males to females). Weiss et al. (2013) highlighted that the sex pheromone of *L. heterotoma* is mainly composed of (−)‐irodomyrmecin (i.e., a type of monoterpenoids), a highly volatile compound produced by female wasps. Four additional minor iridoid components, ((+)‐isoiridomyrmecin, two irodials and a third stereoisomer of iridomyrmecin), appear to be essential for the sex pheromone to be completely bioactive and highly attractive to males (Weiss, Hofferberth, et al., 2015, Weiss et al., 2013; Table 3). These compounds are produced and stored in a cephalic gland, more specifically in a pair of mandibular glands (Stökl et al., 2012; Stökl & Herzner, 2016). A recent study with several *Leptopilina* species, including *L. heterotoma*, tested the attraction of males towards patches with the odor of the opposite sex or the odor of hosts (Böttinger & Stökl, 2020). Males were only attracted to patches if females were present and were not attracted by host odors (living *Drosophila* larvae on the host patch). Females were more attracted to patches containing host odors than to conspecific male odors, irrespective of their mating status (virgin or mated; Böttinger & Stökl, 2020). This result is consistent with earlier studies showing that *L. heterotoma* females can eavesdrop on adult *Drosophila* pheromone communication, to which females are attracted to locate larval laying sites (Wertheim et al., 2003; Wiskerke et al., 1993).

**TABLE 3:** List of iridoid compounds produced by *L. heterotoma* males and females (a), with or without ant predator attack (b), in mated and virgin females (c), and mate attraction quantities (d).

	Mean amount in ng (±1 *SE*)		Reference
	Male	Female	
a. Iridoid compounds found in *L. heterotoma*			
(−)‐iridomyrmecin	–	236.3 ± 20.6/110.1 ± 16.6	Stökl et al. (2012), Weiss, Hofferberth, et al. (2015)
+)‐isoiridomyrmecin	39.4 ± 3.8	22.1 ± 4.8/5.8 ± 3	Stökl et al. (2012), Weiss, Hofferberth, et al. (2015)
Iridodial 1	Trace	26.1 ± 3.3/10.9 ± 2.8	Stökl et al. (2012), Weiss, Hofferberth, et al. (2015)
Iridodial 2	–	9.1 ± 0.8/5 ± 1.6	Stökl et al. (2012), Weiss, Hofferberth, et al. (2015)
Third stereoisomere of iridomyrmecin	–	4.9 ± 1.2	Stökl et al. (2012), Weiss, Hofferberth, et al. (2015)
b. Total amount of iridomyrcecins released by *L. heterotoma*			
Females (10), not attacked	3 ± 1.2		Stökl et al. (2012)
Females (10), attacked by *Myrmica rubra*	370 ± 70		Stökl et al. (2012)
Females (3), attacked by *Cardiocondyla obscurior*	Trace		Stökl et al. (2015)
Females (3), attacked by *M. scabrinodis*	18.2*		Stökl et al. (2015)
Males (10), not attacked	5.8 ± 2.2		Stökl et al. (2012)
Males (10), attacked by *M. rubra*	61.8 ± 20.6		Stökl et al. (2012)
c. Total amount of (−)‐iridomyrmecin released by *L. heterotoma*			
Mated females (10)	3 ± 1.2		Stökl et al. (2012), Weiss et al. (2013)
Virgin females (10)	15.5*		Weiss et al. (2013)
d. Amount of *L. heterotoma* female iridomyrmecins (in ng)	Attraction of males		
60	Yes		Weiss et al. (2013)
30	Yes		Weiss et al. (2013)
15	Yes		Weiss et al. (2013)
8	Yes		Weiss et al. (2013)
4	No		Weiss et al. (2013)

*Note*: The mean (±1 *SE*) or median (*) amounts are provided in ng based on reported values in the cited references.

Sex pheromones can also play a key role for species recognition and mate choice on a short range. Within the *Leptopilina* genus, females of most species, including *L. victoriae* (Weiss, Ruther, et al., 2015), *L. clavipes* (Pfeiffer et al., 2018) and *L. ryukyuensis* (Böttinger et al., 2019) rely on cuticular hydrocarbons and/or iridoids, to attract males. In contrast, *L. heterotoma* solely relies on iridoids (Böttinger et al., 2021). The specificity of mate attraction in *L. heterotoma* remains unclear, however, as contrasting results have been obtained (Fauvergue et al., 1999; Weiss et al., 2013). The question is whether males are still able to discriminate against heterospecifics during courtship. To test this, Weiss et al. (2013) compared wing fanning times (i.e., part of the courtship sequence; see Section 7 on mating behaviors) of *L. heterotoma* males when presented with paper filters impregnated with either *L. heterotoma* or *L. boulardi* female iridoids. Male wing fanning lasted significantly longer when *L. heterotoma* males were exposed to iridoids from conspecific females, meaning that males recognized conspecific females and were prepared to mate. Mate recognition (as opposed to mate attraction) is thus species‐specific and mediated by a blend of iridoid compounds characteristic for *L. heterotoma* (Weiss et al., 2013; Weiss, Hofferberth, et al., 2015; Weiss, Ruther, et al., 2015).

Sex pheromone communication was proposed to have evolved from precursor molecules initially used for other purposes, i.e., the sender‐precursor hypothesis (Stökl & Steiger, 2017; Wyatt, 2014). This hypothesis states that any compound released by one individual and detected by another individual of the same species can evolve into a sex pheromone if there is a selective advantage for both sender and receiver (Wyatt, 2014). Chemical compounds are generally synthesized in limited quantities and assumed to be costly, so reusing existing compounds for chemical communication may be favored by selection, a phenomenon referred to as “semiochemical parsimony” (Blum, 1996). For a long time, data supporting the sender‐precursor hypothesis remained rare in insects, mainly because most studies only experimentally tested a pheromone's function, while neglecting the study of primary functions.

Sex pheromones in *L. heterotoma* have attractive properties, but also seem to be repellent. *L. heterotoma* females indeed emit a defensive secretion composed of (−)‐iridomyrmecin (around 80% of the secretion) and minor amounts of the four other iridoid compounds (Stökl et al., 2012; Weiss et al., 2013). In males, this secretion is composed of a single compound: (+)‐isoiridomyrmecin (Stökl et al., 2012). The pheromone secretion is released during an attack from natural enemies, such as ants, but in much higher quantities compared to use as sex pheromones (Stökl et al., 2012, 2015; Table 3). Due to the larger size of female *L. heterotoma* mandibular glands, females can release larger amounts of iridoid compounds than males (Stökl & Herzner, 2016). Females are also able to discriminate between predator species and to control and adjust the amount of iridoids to release accordingly (Stökl et al., 2012, 2015; Stökl & Herzner, 2016; Table 3).

The threefold use of (−)‐iridomyrmecin by *L. heterotoma* as sex pheromone, for defense, and competition avoidance (see Section 5 on competitive interactions; Weiss et al., 2013), represents an example of a semiochemical parsimony that reinforces the sender‐precursor hypothesis (Stökl & Steiger, 2017; Wyatt, 2014). The use of (−)‐iridomyrmecin might have evolved from a defensive compound to a competition avoidance cue to a female sex pheromone (Stökl & Steiger, 2017). In this context, the costs and benefits for males responding to iridoids must be evaluated, because (−)‐iridomyrmecin attraction can both increase the probability of finding a female and thus mating success, but at a risk of being harmed by a predator if the defensive chemical compounds released by the female are not sufficient to repulse it. Assessing predation risk and the use of defensive compounds in natural populations or recently field‐caught *Leptopilina* wasps would help to determine the selective pressure on males to better understand the evolution of sex pheromones.

## 7 MATING‐RELATED TRAITS AND POPULATION STRUCTURING

Mate finding, dispersal, and mate choice decisions can have major evolutionary consequences that have often been studied in parasitoids by examining patterns of sex allocation (Hardy, 1994). Key theoretical advancements were made by Fisher's frequency dependent selection for equal sex ratios (Fisher, 1930), and Hamilton's local mate competition theory predicting female‐biased sex ratios because related males compete for mates (Hamilton, 1967). Depending on the system, the ecology of mating can lead to clear population structuring (local mating) or panmixis (random mating) at the extremes, although intermediate mating structures, such as partial local mating, may actually be most common (Hardy, 1994). In this section, we look at research concerned with mate‐finding, dispersal, mating, and sex ratio distortion in the amber wasp *L. heterotoma*.

In many animals, mate finding is a crucial step for producing viable offspring, but in haplodiploids, such as Hymenoptera, mating is not a necessity (Cook, 1993; Godfray, 1988, 1990; Godfray & Grafen, 1988; Hardy, 1994). In haplodiploids, including *L. heterotoma*, unfertilized eggs develop into haploid males and fertilized eggs into diploid females (Heimpel & de Boer, 2008). Virgin females are thus able to reproduce, but generate exclusively male offspring (so‐called “constrained sex allocation”; Godfray, 1990), whereas mated females can control the sex ratio of offspring by choosing whether to fertilize an egg before oviposition or not. Another consequence of haplodiploidy is that virgin females face a trade‐off between mate‐searching (to be able to produce daughters) and host‐searching (to immediately produce sons only; Godfray, 1990). In contrast to female reproductive success (e.g., the number of eggs produced), male reproductive success depends on the number of fertile females he can mate with, leading to distinct reproductive strategies for both sexes.

### 7.1 Dispersal

In a recent study, Böttinger and Stökl (2020) investigated mate finding and dispersal from the natal patch in males and females of four *Leptopilina* species, including *L. heterotoma*. On average, *L. heterotoma* males emerged about 2 days before females (but see Eijsackers and Bakker (1971) and Fauvergue et al. (1999) showing within‐brood emergence is similar for males and females). This daily rhythm could be an adaptation to competition between males, as the first emerging males can court and mate with more females (Fagerström & Wiklund, 1982; Fauvergue et al., 1999; Pompanon et al., 1995). Dispersal of both males and females occurred directly after emergence from the natal patch. Males thus start dispersing before conspecific females emerge on the same patch. Fauvergue et al. (1999) indeed already showed that about 20% of both males and females emerged without a potential mate present. Moreover, dispersal of *L. heterotoma* females was up to three times higher compared to the other *Leptopilina* species, where a similar proportion of males and females dispersed (Böttinger & Stökl, 2020; Fauvergue et al., 1999). Individuals that emerge (and disperse) in the absence of conspecifics may favor off‐patch matings and reduce local mate‐competition, but on the other hand may compete with males present on another patch. Dispersal of *L. heterotoma* thus appears to differ from other *Leptopilina* species and other parasitoid wasp species, where males wait for the emergence of conspecific females to mate on the natal patch (Carton et al., 1986; Godfray, 1994; Godfray & Hardy, 1990). Post‐emergence dispersal of *L. heterotoma* males appears to be beneficial and does not pose a risk in finding mating partners.

Variation in dispersal within the *Leptopilina* genus was recently found to be related to chemical compounds released by females (Böttinger & Stökl, 2020). The volatility of sex pheromones can be an important determinant of male and female wasp dispersal behavior (Böttinger & Stökl, 2020). For *Leptopilina* species that use highly volatile sex pheromones (i.e., iridoids; see Section 6 on chemical communication), such as *L. heterotoma* or *L. japonica*, males started to disperse immediately after emergence and the presence of females does not affect the dispersal rate (Böttinger & Stökl, 2020). *L. heterotoma* females also showed a significantly higher dispersal rate compared to heterospecific females emitting sex pheromones that are less volatile. Furthermore, whether hosts are present or not can affect dispersal propensity. *L. heterotoma* males were more attracted to patches with females, whereas virgin and mated females dispersed towards patches containing host odors (Böttinger & Stökl, 2020). Moreover, *L. heterotoma* males were found to be attracted to volatiles emitted only by their conspecific virgin females (i.e., there was no attraction to mated females) both in the field and in the laboratory (Fauvergue et al., 1999). Based on similar findings in another wasp (*Lysiphlebus testaceipes*), we can hypothesize that *L. heterotoma* virgin females are able to search for hosts while emitting sex pheromones to attract males (Fauvergue et al., 2008). Dispersal of males would then be driven solely by mate‐searching (and feeding), which is indeed easier for species that emit highly volatile iridoid sex pheromones, such as *L. heterotoma*. The high dispersal rate of *L. heterotoma* males may increase their mating opportunities and success, as they can mate several times in nature, while females seem to mate only once (see below). Although nothing is known about competition between *L. heterotoma* conspecific males for mating opportunities, we can assume that the high dispersal rate also decreases fights among male wasps (Godfray, 1994). Such a strategy, where males disperse rapidly from the natal patch in search of females guided by volatile pheromones deviates from expectations under local mate competition in haplodiploid species (Hamilton, 1967; Hardy, 1994).

Parasitoids can lay a single (solitary) or multiple eggs (gregarious) inside a single host. When hosts are aggregated on patches, however, solitary parasitoids can be considered “quasi‐gregarious”. *L. heterotoma* is indeed quasi‐gregarious, due to the high aggregation of *Drosophila* larvae on single patches (Fauvergue et al., 1999). Mating in *L. heterotoma* was assumed to be restricted to a local patch, where brothers compete for females, leading to strict local mate competition and female‐biased offspring sex ratios (Hamilton, 1967). The mating system of some parasitoid species, including *L. heterotoma*, does, however, not seem to follow Hamilton's predictions (Fauvergue et al., 1999; Hardy, 1994). Reviewing the mating structure of 22 parasitoid species, Hardy (1994) concluded that complete local mating is exceptional, rather than the norm in gregarious and quasi‐gregarious parasitoids. Moreover, Fauvergue et al. (1999) reviewed the literature on long‐distance volatile sex pheromones in parasitoids and found that 21 species, including *L. heterotoma*, use sex pheromones for mate finding, including gregarious, quasi‐gregarious and solitary species. Volatile sex pheromones aim to facilitate dispersal and off‐patch matings, which in turn reduce local mate competition, sib‐mating, the risk of inbreeding, and competition between males. The conclusions of Böttinger and Stökl (2020) align well with the suggestion of Hardy (1994) and results of Fauvergue et al. (1999) that *L. heterotoma* shows partial local mate competition, with both on‐patch and off‐patch mating. These observations reinforce the conclusion that off‐patch mating may be frequent in gregarious and quasi‐gregarious parasitoids, but more data is needed to develop hypotheses on the evolution of such mating structures.

### 7.2 Courtship and mating

Once a potential mate has been located, *L. heterotoma* shows a stereotypical courtship sequence, like many other insects (described in more details in Isidoro et al., 1999; van den Assem, 1969). Both males and females are sexually receptive immediately after emergence. Courtship starts with the male rapidly fanning (i.e., vibrating) his wings, without making actual contact with the female. While wing fanning takes place, the male will position his antennae forward and will start following the female. Once the male is in close enough proximity, he will make physical contact with the female, initially only with his antennae. He will then attempt to mount the female and place his antennae parallel with those of the female. The male will then ‘paddle’ the club‐shaped part of the female's antennae with his own antennae. A receptive female will subsequently extrude her ovipositor to expose her genital aperture. The male then ceases wing fanning and paddling, moves backwards and spreads his wings before copulating with the female, which typically requires more than one attempt. Once the male dismounts, both male and female will start preening different body parts, while the female will again conceal her genitalia. The male may attempt a new courtship sequence, but a female will generally not mate more than once, at least not in the laboratory (van den Assem, 1969).

If a *L. heterotoma* male is unsuccessful in copulating with a female despite several attempts, the male will dismount while the female continues to show typical behaviors observed during copulation (absence of movement; van den Assem, 1969). Such unfertilized females will conceal their genital area after the typical duration of a successful copulation and will not copulate again; hence behaviorally these females respond as if copulation was successful, also called “pseudo‐virgins” (Godfray, 1994). Placing males and females together is thus no guarantee that a female will have successfully mated, although it is not clear how frequently courtship is unsuccessful for males. Unmated females will have suffered at least some of the costs associated with mating (e.g., the cost of being courted, a reduction in time she can dedicate to finding and parasitizing hosts) without having the benefits (to produce female offspring). The question is how common pseudo‐virgins are in the field and whether these females will mate again. Mated females will generally only become receptive again after several weeks in the laboratory (van den Assem, 1969), and sex allocation patterns suggest that sperm may be depleted 6 days after mating as only males are produced (Chabora et al., 1979). Under optimal conditions in the field (e.g., sufficient host availability), multiple mating may thus not be necessary when actual mating has occurred.

Comparing the same *L. heterotoma* strain as van den Assem (1969) with another strain from the USA, Veerkamp (1982) showed that the latter differed considerably in the timing of mating‐related behaviors, offspring numbers, and sex allocation patterns. This would suggest that mating‐related behaviors may depend largely on the local environment, leading to local adaptation and population differentiation. Ridley (1993) suggested that solitary hymenopteran species are primarily monandrous (females mate once), while gregarious species are mainly polyandrous (females mate multiple times). Considering the strong effects of local environmental conditions, different patterns of dispersal observed between populations (described above), and a quasi‐gregarious host distribution, we could expect at least some multiple mating to occur when siblings are competing at the natal patch. This remains strictly hypothetical for *L. heterotoma*, but for other monandrous species, such as *Nasonia vitripennis*, *Aphelinus asychis*, *Trichogramma evanescens* (Boulton et al., 2015, 2019; Damiens & Boivin, 2005; Jacob & Boivin, 2005; Ridley, 1988; Wang et al., 2021), multiple mating is occasionally observed in the field. It would be very interesting to compare mating‐related traits between distinct, natural wasp populations, a task for which *L. heterotoma* is particularly well suited.

### 7.3 *Wolbachia* and cytoplasmic incompatibility

Factors unrelated to mating structure can also have a large effect on sex allocation patterns, including the intracellular alpha‐proteobacterium *Wolbachia pipientis. Wolbachia* is inherited maternally (i.e., vertical transmission), but it can occasionally also be acquired horizontally from a conspecific (Frost et al., 2014) or another species (Ahmed et al., 2016). The density and location of *Wolbachia* inside *L. heterotoma* is sex‐dependent: females harbor a greater number of bacteria per cell compared to males, and in females *Wolbachia* is mainly located in the abdomen compared to the head and thorax in males (Mouton et al., 2003). *L. heterotoma* can harbor three different *Wolbachia* strains, *wLhet1*, *wLhet2* and *wLhet3*, and these strains all belong to the *Wolbachia* A clade (Vavre et al., 1999, 2000; Werren et al., 1995; Zhou et al., 1998). Infection with the three distinct *wLhet* strains appears to predominate in nature, although double‐, mono‐ and non‐infected individuals have also been recorded in natural populations (Mouton, 2004). In contrast to *A. tabida* that requires *Wolbachia* for oogenesis (Mouton et al., 2009), none of the three strains is obligatory for *L. heterotoma* (Vavre et al., 2000). However, *wLhet1* does seem to be required for persistence of the other strains, because mono‐infection with *wLhet2* or *wLhet3* could not be established in the laboratory (Mouton et al., 2003). The density of each *Wolbachia* strain remains constant regardless of the presence of other strains, suggesting an absence of competition between strains (Mouton et al., 2003). The relative proportion of the three strains does not vary depending on temperature or host genotype: *wLhet3* is always the most abundant, while *wLhet2* is the least abundant (Mouton et al., 2003, 2007). Temperature and host genotype do, however, affect the total *Wolbachia* load (Mouton et al., 2007).

**FIGURE 6:**
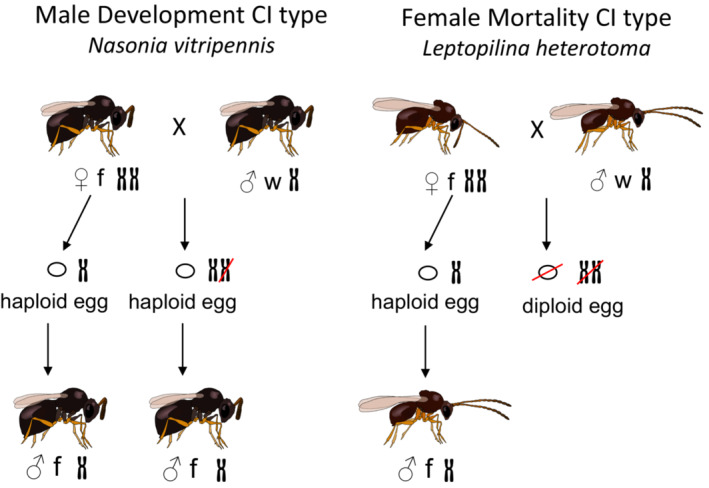
The two types of cytoplasmic incompatibility induced by *Wolbachia* in haplodiploid insects: the Male Development type described in *Nasonia vitripennis* (Breeuwer & Werren, 1990) and the Female Mortality type described in *Leptopilina heterotoma* (Vavre et al., 2000, 2001). f: *Wolbachia*‐free, w: infected with *Wolbachia* (*wNvitA* and *wNvitB* for *N. vitripennis*; *wLhet1*, *wLhet2* and *wLhet3* for *L. heterotoma*).

*Wolbachia* can have various effects on host fitness, including cytoplasmic incompatibility: a reproductive incompatibility resulting in embryonic death (Shropshire et al., 2020). In diploids, where all eggs are fertilized, a complete cytoplasmic incompatibility leads to loss of all progeny. In haplodiploids, two types of cytoplasmic incompatibility have been described: “Female Mortality” and “Male Development” (Figure 6). For Female Mortality cytoplasmic incompatibility, fertilized eggs cannot develop; hence only males are produced from unfertilized eggs (Figure 6). For Male Development cytoplasmic incompatibility, fertilized eggs lose the paternal chromosome and develop into haploid males. While the genes underlying cytoplasmic incompatibility have been discovered, the molecular mechanisms and differences between the two incompatibility types have not yet been elucidated (Shropshire et al., 2020). In general, the incompatibility type and number of offspring resulting from an incompatible cross depends on several factors, such as host species, host genotype, as well as *Wolbachia* strain and *Wolbachia* load (Bordenstein et al., 2003; Raychoudhury & Werren, 2012). In *L. heterotoma*, cytoplasmic incompatibility induced by the three strains (*wLhet1*/*wLhet2/wLhet3* male × *Wolbachia*‐free female) resulted in a Female Mortality type incompatibility (Vavre et al., 2000, 2001). Curiously, crosses between *L. heterotoma* individuals containing only one or two *Wolbachia* strains revealed different types of cytoplasmic incompatibility, intermediate between Female Mortality and Male Development incompatibility, where part of the offspring died, while some developed as haploid males. The percentage of haplodized eggs decreased with the number of strains involved from ~41% to ~18% (Mouton et al., 2005). This clearly indicates that cytoplasmic incompatibility is dependent on the number of strains and/or *Wolbachia* density in *L. heterotoma*. The three *wLhet* strains are further bidirectionally incompatible, meaning that any cross between individuals bearing different strains would result in embryonic death (Mouton et al., 2005).

In addition to the clear negative fitness consequences of cytoplasmic incompatibility, *Wolbachia* can further reduce *L. heterotoma* fitness by reducing locomotor performance, survival under starvation, and egg production (Fleury, Vavre, et al., 2000). This has, however, only been tested with triply infected individuals; hence the effects of each strain on fitness are not known (Fleury, Vavre, et al., 2000). The eggs of *Wolbachia*‐cured females showed a lower encapsulation rate by *D. simulans* larvae, revealing that immunity‐related traits are also likely affected (Fytrou et al., 2006; see Section 2 on host immunity). No effect was found of *Wolbachia* infection on circadian rhythm or development time (Fleury, Vavre, et al., 2000). When both parents were triply infected, sex ratios remained unchanged, indicating that *Wolbachia* is not feminizing nor male‐killing in *L. heterotoma* (Fleury, Vavre, et al., 2000). So far, no positive fitness effects of *Wolbachia* have been found for *L. heterotoma* (Fleury, Vavre, et al., 2000). Considering its strong negative effects on fitness and its high prevalence in natural populations, *Wolbachia* is particularly deleterious for *L. heterotoma*.

## 8 KEY FEATURES OF *L. HETEROTOMA* AS A MODEL SYSTEM

The incredible knowledge‐base on the amber wasp *L. heterotoma* described largely in this review highlights the major contribution that this model system has made to research in ecology and evolution. *L. heterotoma* phenotypes often lie in between the most extreme life history syndromes. For example, development occurs as an endo‐ and ectoparasitoid, fat accumulation is plastic and dependent on the host environment, there seems to be partial local mate competition, and cytoplasmic incompatibility involves both types (Male Development and Female Mortality). This makes *L. heterotoma* an excellent system for comparative studies.

Previous work on *L. heterotoma* further paves the way for the development of novel research in different fields. For example, *L. heterotoma* would be an excellent system to determine the cost of learning and the trade‐offs between learning ability and life histories in changing environments. We further know very little about the species' basic population dynamics (e.g., rate of increase, density‐dependence), knowledge that could be of use for linking individual‐level and population‐level processes. There is still a major gap of knowledge on mate choice decisions and sexual selection in *L. heterotoma*, which could play an important role in population differentiation and speciation. Indeed, aside from the work on local mate competition, we still know relatively little about genetic differentiation between populations, including dispersal distance, migration, and gene flow. Sequencing neutral markers revealed large gene flow and minor sequence differences between *L. heterotoma* populations (Visser et al., 2018), but phenotypically we see major intra‐specific differences in diverse traits, such as mating behaviors, egg numbers, and fat accumulation phenotypes.

Recently, a high quality and annotated genome sequence of *L. heterotoma* became available (Di Giovanni et al., 2020; Huang et al., 2021; Wey et al., 2020). The genome was originally sequenced to study the evolution of virus‐like particles, and genomic tools have indeed mostly been used in studies on virulence and immunity (Wertheim, 2022). Genetic tools, such as gene‐targeted knock‐down RNA interference (RNAi) and CRISPR‐Cas9 have been widely used to characterize gene functions, also in parasitoids (Dalla Benetta et al., 2020; Li et al., 2012, 2017; Lynch, 2006; Werren, 2009). A successful RNAi method was already developed for *L. boulardi* by injecting dsRNA directly into dissected late larval instars (Colinet et al., 2014). Using a similar method, a recent study with *L. heterotoma* also showed high RNAi efficiency (Huang et al., 2021). This provides an exciting prospect for future studies on gene function in *L. heterotoma*.

Another asset is *L. heterotoma's* close association with *Drosophila* species, where *D. melanogaster* is itself an important model in genetics, developmental biology and genomics (Gompel & Carroll, 2003; Kuntz & Eisen, 2014; Prud'homme & Gompel, 2010; Ugur et al., 2016). There are thus a plethora of resources available for the host, e.g., mutants, the *Drosophila* Genetic Reference Panel (Mackay et al., 2012), and the more recent literature has increased focus also on *Drosophila* ecology (O'Grady & DeSalle, 2018). Most hosts (Bombin & Reed, 2016; Klepsatel et al., 2018, 2020; Krams et al., 2020; Wertheim et al., 2006) and parasitoids (Fleury et al., 2004, 2009; Mazzetto et al., 2016) are further easily observed and caught in the field, as well as reared in the lab (i.e., using artificial media, short generation times, high offspring numbers). Altogether, this makes the *L. heterotoma*‐*Drosophila* system an excellent eco‐evolutionary model system for studying host‐parasitoid dynamics and interactions, also in natural populations.

## AUTHOR CONTRIBUTIONS

**Maude Quicray:** Validation (equal); writing – original draft (equal); writing – review and editing (equal). **Léonore Wilhelm:** Validation (equal); writing – original draft (equal); writing – review and editing (equal). **Thomas Enriquez:** Validation (equal); writing – original draft (equal); writing – review and editing (equal). **Shulin He:** Validation (equal); writing – original draft (equal). **Mathilde Scheifler:** Validation (equal); writing – original draft (equal); writing – review and editing (equal). **Bertanne Visser:** Conceptualization (lead); funding acquisition (lead); project administration (lead); supervision (lead); validation (equal); writing – original draft (equal); writing – review and editing (equal).

## CONFLICT OF INTEREST

The authors declare no conflict of interest.

## Supporting information


Table S1.

Table S2.

## Data Availability

Data sharing not applicable to this article as no datasets were generated or analysed during the current study.
