# Supplementary material for: The Drosophila‐parasitizing wasp Leptopilina heterotoma: A comprehensive model system in ecology and evolution
Source: Ecol Evol. 2023 Jan 23;13(1):e9625. doi: 10.1002/ece3.9625 (PMC9871341; doi:10.1002/ece3.9625)
Supplement: Supplementary file 2 — Table S2. [file ECE3-13-e9625-s002.pdf]

The *Drosophila*-parasitizing wasp *Leptopilina heterotoma*:

A comprehensive model system in ecology and evolution

Maude Quicray*, Léonore Wilhelm*, Thomas Enriquez, Shulin He, Mathilde Scheifler & Bertanne Visser

*co-first authors

Evolution and Ecophysiology group, Department of Functional and Evolutionary Entomology, University of Liège - Gembloux Agro-Bio Tech, Passage des Déportés 2, 5030 Gembloux, Belgium

Supplementary table 2. *The ability of L. heterotoma to parasitize and develop on different Drosophila species, including an estimate of the percentage survival on each host based on data available in the literature. The substrate on which the hosts lay eggs is also reported.*

**Host species**	**Group**	**Host substrate**	**Successful development?**	**Survival**	**References**
D. (Dorsilopha) busckii	Dorsilopha	Vegetables (cauliflower, lettuce, zucchini), potatoes, fruits (melon)	No	0%	Atkinson and Shorrocks, 1977; Girod, Lierhmann, *et al.*, 2018
D. funebris	Funebris	Fruit, Other plant parts, Fungus	Yes	NA	Markow and O'Grady, 2008; Trivellone *et al.*, 2020
D. immigrans	Immigrans	Cactus, Other plant parts, Flux, Fungus, Fruits	Low	2%	Janssen, 1989; Markow and O'Grady, 2008

			Low	<1%	Girod, Lierhmann, *et al.*, 2018
			Yes	19%	Hedlund, Vet and Dicke, 1996
D. ananassae	Melanogaster	Fruits, flowers and fungus	No	0%	Buruga and Olembo, 1971; Schlenke *et al.*, 2007
D. biarmipes	Melanogaster	Rotten fruits	No	0%	Schlenke *et al.*, 2007; Atallah *et al.*, 2014; Dweck *et al.*, 2021
D. erecta	Melanogaster	*Pandanus* fruits	Yes	59.7%	Lachaise *et al.*, 1988; Lynch, Schlenke and Roode, 2016
			Yes	41%	Schlenke *et al.*, 2007
D. eugracilis	Melanogaster	Fruits (banana traps)	Yes	46%	Schlenke *et al.*, 2007; Kimura and Suwito, 2012
D. lutescens	Melanogaster	Fruits and flowers	Low	1%	Schlenke *et al.*, 2007; Mitsui, Beppu and Kimura, 2010
D. mauritiana	Melanogaster	Fruits	Yes	76%	Schlenke *et al.*, 2007
D. melanogaster	Melanogaster	Fruit, Flower, Other plant parts, Cactus	Yes	26.1%	Janssen, 1989; Markow and O'Grady, 2008
			Yes	89.5%	Lynch, Schlenke and Roode, 2016
			Yes	93%	Schlenke *et al.*, 2007
			Yes	88.6%	Carton *et al.*, 1991
			Yes	46.7%	Girod, Lierhmann, *et al.*, 2018
D. orena	Melanogaster	Waterberry (*Syzygium staudtii*)	Yes	80.9%	Lynch, Schlenke and Roode, 2016; Comeault *et al.*, 2017
D. paralutea	Melanogaster		No	0%	Schlenke *et al.*, 2007
D. santomea	Melanogaster		Yes	42.9%	Lynch, Schlenke and Roode, 2016
				61%	Schlenke *et al.*, 2007
D. sechellia	Melanogaster	Fruits of *Morinda citrifolia* (Rubiaceae) specifically	Yes	64%	Lachaise *et al.*, 1988; R'Kha, Capy and David, 1991; Schlenke *et al.*, 2007

D. simulans	Melanogaster	Fruits	Yes	65.3%	Janssen, 1989
			Yes	78%	Lynch, Schlenke and Roode, 2016
			Yes	56%	Schlenke *et al.*, 2007
			Yes	51%	Carton *et al.*, 1991
D. suzukii	Melanogaster	Flowers and ripe fruits	No	0%	Mitsui, Beppu and Kimura, 2010; Lynch, Schlenke and Roode, 2016; Dweck *et al.*, 2021
					Poyet *et al.*, 2013; Lynch, Schlenke and Roode, 2016; Mazzetto *et al.*, 2016, 2016; Trivellone *et al.*, 2020
			Low	<1%	Rossi-Stacconi *et al.*, 2015; Girod, Lierhmann, *et al.*, 2018
D. teissieri	Melanogaster	*Psidium guajava*, *Ficus* sp., *Parinari excelsa*, *Citrus maxima*, *Tieghemella heckelii*	Yes	31.4%	Lachaise *et al.*, 1988; Lynch, Schlenke and Roode, 2016
				64%	Schlenke *et al.*, 2007
D. suboscura	Obscura	Fruits and Sap-fluxes	Yes	87.3%	Janssen, 1989
			Yes	30.4%	Girod, Lierhmann, *et al.*, 2018
D. obscura	Obscura	Fruits and Sap-fluxes	Yes	17.5%	Janssen, 1989
D. pseudoobscura	Obscura	Fruits and Sap-fluxes	Yes	90%	Dobzhansky and Epling, 1944; Carson, 1951; Schlenke *et al.*, 2007
D. tristis	Obscura	Fruits	Yes	5.6%	Janssen, 1989
D. kuntzei	Quinaria	Sap-fluxes	Yes	89.3%	Janssen, 1989
D. phalerata	Quinaria	Fungus, Fruits and Sap-fluxes	Yes	49.5%	Janssen, 1989; Driessen, Hemerik and van Alphen, 1990

D. buzzati	Repleta	Tissues of Opuntieae, Trichocereinae and Cereae cacti	Yes	34.6%	Carson and Wasserman, 1965; Ruiz, Fontdevila and Wasserman, 1982; Carton *et al.*, 1991; Hasson, Naveira and Fontdevila, 1992; Fanara, Fontdevila and Hasson, 1999
D. hydei	Repleta	Tissues and fruits of *Opuntia* cacti	Yes	>90% (6/9 strains) 56-82% (3/9 strains)	Xie, Vilchez and Mateos, 2010; Oliveira *et al.*, 2012
D.robusta	Robusta	Flux	Yes	30%	Schlenke *et al.*, 2007; Markow and O'Grady, 2008
D. littoralis	Virilis	Fruits and Sap-fluxes	Yes	10.8%	Janssen, 1989
D. virilis	Virilis	Flux	Yes	59%	Schlenke *et al.*, 2007; Markow and O'Grady, 2008
D. willistoni	Willistoni	Flower, Fruit	Yes	71%	Schlenke *et al.*, 2007; Markow and O'Grady, 2008
Zaprionus vittiger	Zaprionus (armatus group)	Flower, Fruit	Low	4%	Buruga and Olembo, 1971; Buruga, 1976; Schlenke *et al.*, 2007

References

Atallah, J., Teixeira, L., Salazar, R., Zaragoza, G., Kopp, A., 2014. The making of a pest: the evolution of a fruit-penetrating ovipositor in *Drosophila suzukii* and related species. *Proceedings of the Royal Society B*, 281, p.20132840. https://doi.org/10.1098/rspb.2013.2840

Atkinson, W., Shorrocks, B., 1977. Breeding site specificity in the domestic species of *Drosophila*. *Oecologia*, 29, p.223–232. https://doi.org/10.1007/BF00345697

Buruga, J.H., 1976. Breeding sites of somes species of *Zaprionus* (Diptera) in Uganda. *Jurnal of the East Africa Natural History Society and National Museum,* 31, p.1-6.

Buruga, J.H., Olembo, R.J., 1971. Plant food preferences of some sympatric Drosophilids of Tropical Africa. *Biotropica*, 3(2), p.151-158. https://doi.org/10.2307/2989820

Carson, H.L., 1951. Breeding sites of *Drosophila pseudoobscura* and *Drosophila persimilis* in the transition zone of the Sierra Nevada. *Evolution*, 5(2), p.91–96.

Carson, H.L., Wasserman, M., 1965. A widespread chromosomal polymorphism in a widespread species, *Drosophila buzzatii*. *American Naturalist*, 99(905), p.111–115. https://doi.org/10.1086/282356

Carton, Y., Haouas, S., Marrakchi, M., Hochberg, M., 1991. Two competing parasitoid species coexist in sympatry. *Oikos*, 60(2), p.222-230. https://doi.org/10.2307/3544869

Comeault, A.A., Serrato-Capuchina, A., Turissini, D.A., McLaughlin, P.J., David, J.R., Matute, D.R., 2017. A nonrandom subset of olfactory genes is associated with host preference in the fruit fly *Drosophila orena*: behavioural specialization in *D. Orena*. *Evolution Letters,* 1(2), p.73–85. https://doi.org/10.1002/evl3.7

Dobzhansky, T., Epling, C., 1944. Contributions to the genetics, taxonomy, and ecology of *Drosophila pseudoobscura* and its relatives. *Carnegie Institution of Washington Publication,* 554*,* p.183*,* Washington, D. C*.*

Driessen, G., Hemerik, L., van Alphen, J., 1990. *Drosophila* species, breeding in the stinkhorn (*Phallus impudicus* Pers.) and their larval parasitoids. *Netherlands Journal of Zoology,* 40(3), p.409–427. https://doi.org/10.1163/156854290x00019

Dweck, H.K., Talross, G.J., Wang, W., Carlson, J.R., 2021. Evolutionary shifts in taste coding in the fruit pest *Drosophila suzukii*. *eLife*, 10, p.e64317. https://doi.org/10.7554/eLife.64317

Fanara, J.J., Fontdevila, A., Hasson, E., 1999. Oviposition preference and life history traits in cactophilic *Drosophila koepferae* and *D. buzzatii* in association with their natural hosts. *Evolutionary Ecology,* 13, p.173–190. https://doi.org/10.1023/A:1006636025623

Girod, P., Lierhmann, O., Urvois, T., Turlings, T.C.J., Kenis, M., Haye, T., 2018. Host specificity of Asian parasitoids for potential classical biological control of *Drosophila suzukii*. *Journal of Pest Science,* 91, p.1241–1250. https://doi.org/10.1007/s10340-018-1003-z

Hasson, E., Naveira, H., Fontdevila, A., 1992. The breeding sites of Argentinian cactophilic species of the *Drosophila mulleri* complex (subgenus *Drosophila*-repleta group). *Revista Chilena de Historia Natural,* 65, p.319–326.

Hedlund, K., Vet, L.E.M., Dicke, M., 1996. Generalist and specialist parasitoid strategies of using odours of adult drosophilid flies when searching for larval hosts. *Oikos*, 77(3), p.390-398. https://doi.org/10.2307/3545929

Janssen, A., 1989. Optimal host selection by *Drosophila* parasitoids in the field. *Functional Ecology*, 3(4), p.469–479. https://doi.org/10.2307/2389621

Kimura, M.T., Suwito, A., 2012. Diversity and abundance of frugivorous drosophilids and their parasitoids in Bogor, Indonesia. *Journal of Natural History*, 46(31-32), p.1947–1957. https://doi.org/10.1080/00222933.2012.707239

Lachaise, D., Cariou, M., Lemeunier, F., Tsacas, L., Ashburner, M., 1988. Historical biogeography of the *Drosophila melanogaster* species subgroup. *Evolutionary Biology,* 22, p.159-225. https://doi.org/10.1007/978-1-4613-0931-4_4

Lynch, Z.R., Schlenke, T.A., Roode, J.C., 2016. Evolution of behavioural and cellular defences against parasitoid wasps in the *Drosophila melanogaster* subgroup. *Journal of Evolutionary Biology,* 29(5), p.1016–1029. https://doi.org/10.1111/jeb.12842

Markow, T.A., O'Grady, P., 2008. Reproductive ecology of *Drosophila*. *Functional Ecology*, 22(5), p.747–759. https://doi.org/10.1111/j.1365-2435.2008.01457.x

Mazzetto, F., Marchetti, E., Amiresmaeili, N., Sacco, D., Francati, S., Jucker, C., Dindo, M.L., Lupi, D., Tavella, L., 2016. *Drosophila* parasitoids in northern Italy and their potential to attack the exotic pest *Drosophila suzukii*. *Journal of Pest Science,* 89, p.837–850. https://doi.org/10.1007/s10340-016-0746-7

Mitsui, H., Beppu, K., Kimura, M.T., 2010. Seasonal life cycles and resource uses of flower- and fruit-feeding drosophilid flies (Diptera: Drosophilidae) in central Japan. *Entomological Science,* 13(1), p.60–67. https://doi.org/10.1111/j.1479-8298.2010.00372.x

Oliveira, D.C.S.G., Almeida, F.C., O'Grady, P.M., Armella, M.A., DeSalle, R., Etges, W.J., 2012. Monophyly, divergence times, and evolution of host plant use inferred from a revised phylogeny of the *Drosophila repleta* species group. *Molecular Phylogenetics and Evolution,* 64(3), p.533–544. https://doi.org/10.1016/j.ympev.2012.05.012

Poyet, M., Havard, S., Prevost, G., Chabrerie, O., Doury, G., Gibert, P., Eslin, P., 2013. Resistance of *Drosophila suzukii* to the larval parasitoids *Leptopilina heterotoma* and *Asobara japonica* is related to haemocyte load. *Physiological Entomology,* 38(1), p.45–53. https://doi.org/10.1111/phen.12002

R'Kha, S., Capy, P., David, J.R., 1991. Host-plant specialization in the *Drosophila melanogaster* species complex: a physiological, behavioral, and genetical analysis. *Proceedings of the National Academy of Sciences,* 88(5), p.1835–1839. https://doi.org/10.1073/pnas.88.5.1835

Rossi Stacconi, M.V., Buffington, M., Daane, K.M., Dalton, D.T., Grassi, A., Kaçar, G., Miller, B., Miller, J.C., Baser, N., Ioriatti, C., Walton, V.M., Wiman, N.G., Wang, X., Anfora, G., 2015. Host stage preference, efficacy and fecundity of parasitoids attacking *Drosophila suzukii* in newly invaded areas. *Biological Control,* 84, p.28–35. https://doi.org/10.1016/j.biocontrol.2015.02.003

Ruiz, A., Fontdevila, A., Wasserman, M., 1982. The evolutionary history of *Drosophila buzzatii* III. Cytogenetic relationships between two sibling species of the buzzatii cluster. *Genetics*, 101, p.503–518.

Schlenke, T. a, Morales, J., Govind, S., Clark, A.G., 2007. Contrasting infection strategies in generalist and specialist wasp parasitoids of *Drosophila melanogaster*. *PLoS Pathogens,* 3(10), p.e158. https://doi:10.1371/journal.ppat.0030158

Trivellone, V., Meier, M., Cara, C., Pollini Paltrinieri, L., Gugerli, F., Moretti, M., Wolf, S., Collatz, J., 2020. Multiscale determinants drive parasitization of drosophilidae by Hymenopteran parasitoids in agricultural landscapes. *Insects*, 11(6), p.334. https://doi.org/10.3390/insects11060334

Xie, J., Vilchez, I., Mateos, M., 2010. *Spiroplasma* bacteria enhance survival of *Drosophila hydei* attacked by the parasitic wasp *Leptopilina heterotoma*. *PLoS ONE*, 5(8), p.e12149. https://doi.org/10.1371/journal.pone.0012149
